# Infant and Family Outcomes and Experiences Related to Family-Centered Care Interventions in the NICU: A Systematic Review

**DOI:** 10.3390/children12030290

**Published:** 2025-02-26

**Authors:** Christine R. Hodgson, Renee Mehra, Linda S. Franck

**Affiliations:** Family Health Care Nursing, School of Nursing, University of California, San Francisco, CA 94143, USA

**Keywords:** systematic review, family-centered care, neonatal intensive care, neonate, infant, hospitalization, parent satisfaction, parent–staff relationships

## Abstract

Background/Objectives: Family-centered care (FCC) has been promoted as a model of care in neonatal intensive care units (NICU) for decades. We aimed to synthesize recent evidence about infant and parent outcomes and experiences of FCC interventions in the NICU. Methods: Studies were identified through searches of PubMed, CINAHL, Embase, PsycInfo, and Web of Science (2019–2024). We systematically reviewed English language research from peer-reviewed journals. We excluded studies about Family-Integrated Care and Close Collaboration with Parents to avoid redundancy with other recent reviews. Results: Twenty studies evaluated 19 FCC interventions compared with regular NICU care for a total of 3502 parents/primary caregivers of infants in NICUs in 11 countries. The designs were quantitative (n = 16), qualitative (n = 3), and mixed methods (n = 1). Significant improvements in infant outcomes included feeding (n = 3), weight (n = 1), and length of stay (n = 1). There were no worsened infant outcomes. Significant improvements in parent outcomes included participation, experience, satisfaction, and parent mental health. Two studies had mixed results for parent outcomes, with minimal worsened outcomes. Qualitative findings were also mostly positive. Conclusions: Recent research shows positive infant and family outcomes with a range of FCC interventions in the NICU. However, more RCTs comparing interventions and using similar validated outcome measures are needed.

## 1. Introduction

Family-centered care (FCC) has been a focus of neonatal intensive care unit (NICU) practice, research, and policy for decades [1]. In FCC, children’s parents or other primary caregivers are respected as full partners in healthcare delivery, are actively involved in their children’s care and decision-making, and are engaged in healthcare design and delivery at the organizational level [2]. The movement toward FCC gained momentum as it became apparent that professional caregiving and technology were insufficient to optimize infant outcomes, and that family partnership was crucial to ensure survival and optimize outcomes across low- and high-resource settings [3,4,5,6]. The crucial role of parents’ unique contributions to the care of preterm or ill term infants, such as skin-to-skin care (SSC), human milk, and developmentally supportive care, is well documented, as are the long-term benefits of early parental engagement in infant caregiving and shared decision-making [7,8].

Identifying the most effective FCC interventions and implementing them at scale across diverse neonatal units globally has proven a difficult challenge. It is hampered by a lack of consensus on the most appropriate outcomes and limited research, often conducted with weak study designs [9]. Despite the challenges, research on FCC interventions has grown rapidly over the past 15 years. Two comprehensive models of NICU care delivery that include strong partnerships with former and current NICU families in the design and delivery of care now have a strong evidence base. The Family-Integrated Care (FICare) model includes four fundamental ‘pillars’: educating and supporting healthcare professionals, educating parents, creating a nurturing environment for infants, and providing psychosocial support to families. FICare has been evaluated in the largest neonatal FCC trial to date and has demonstrated a range of improved infant outcomes, such as breastfeeding, weight gain, fewer infections, a shorter duration of supplemental oxygen use and conventional mechanical ventilation, a decreased length of hospital stay (LOS), and improved neurodevelopment compared with usual, unstructured, FCC care delivery across a range of diverse high-, middle-, and low-income settings [1,3]. Another integrated FCC model with strong evidence for effectiveness is the Close Collaboration with Parents (CPP) program [10], which emphasizes education and support for NICU healthcare professionals and focuses on the partner relationship between NICU professionals and parents in infant care and decision-making. CCP has been demonstrated to decrease the LOS, emergency room visits for infants, and maternal depression symptoms and increase parent presence, SSC, and FCC structures overall compared to the usual, unstructured FCC [11,12].

Evidence about other FCC-related interventions has also grown, with several reviews evaluating studies published between 2017 and 2023. Two reviews summarized the diversity in outcomes measured in FCC studies [13,14], one scoping review evaluated FCC principles [15], and one bibliometric review described the growth of FCC studies over time [16]. Some reviews focused on a specific or related aspect of FCC, such as family engagement interventions [17], parent–infant closeness [18], or developmental care [8]. One integrative review evaluated FCC intervention studies conducted across a range of neonatal and pediatric inpatient settings in low- and middle-income countries. However, the neonatal findings were not separately summarized, and the three neonatal studies were included in other systematic reviews [4]. One narrative systematic review evaluated FCC intervention studies conducted across neonatal and pediatric intensive care settings and reported that parent participation and parent–staff collaboration improved parent satisfaction and infant LOS in the NICU setting [19].

Three systematic reviews with meta-analyses of FCC interventions in the NICU that evaluated infant and parent outcomes are particularly noteworthy [5,20,21]. Ding et al. [20] systematically reviewed 19 studies of FCC interventions for preterm infants and their families published in English or Chinese prior to August 2018, resulting in data for 4478 preterm infants and 3158 parents. Nine studies were conducted in China, two in Taiwan, two in the US, and the others in Denmark, Sweden, UK, Iran, India, and Canada/Australia/New Zealand, respectively. The most common FCC intervention evaluated was education support for parents (n = 16 studies), followed by information and communication, personalized care, and parent support or NICU environment interventions. The meta-analysis conducted on 15 of these studies demonstrated significantly increased weight gain and reduced readmission rates for infants receiving FCC interventions compared to those receiving usual care. However, no statistically significant differences were found in neurobehavioral development or the LOS for infants in FCC interventions compared to the usual care groups. The review found significant improvements in parents’ satisfaction, knowledge, and skills, along with reduced levels of stress, anxiety, and depression, for parents receiving FCC interventions compared to parents receiving usual care.

North et al. [21] performed a systematic review and meta-analysis of 15 randomized control trials (RCTs) evaluating family involvement interventions in NICUs for preterm and low-birth-weight (LBW) infants and their families. Studies were included from inception through August 2021 and included a total of 5240 infants, all in middle-income or high-income countries. A meta-analysis of these 15 studies found a significant decrease in retinopathy of prematurity (ROP) and LOS, as well as significant increases in weight gain velocity, neurobehavioral exam scores, and breastmilk intake for infants receiving FCC interventions compared to those receiving usual care. Other findings included decreased infant rates of bronchopulmonary dysplasia (BPD), infection, and intraventricular hemorrhage (IVH), but these studies were of low quality and the findings should be viewed with caution. There were no differences in the mortality or necrotizing enterocolitis (NEC) rates between infants receiving FCC interventions and those receiving usual care. Parents receiving the FCC intervention had significantly lower scores on stress and anxiety measures than parents with usual care.

Reiter et al. [5] systematically reviewed 18 studies evaluating parents in NICU FCC interventions from seven low- and middle-income countries published between inception and February 2022. Meta-analyses were performed for the following outcomes: LOS, mortality, readmission, breastfeeding, growth, development, and parental well-being. The results revealed that parental participation was associated with significantly increased breastfeeding, decreased mortality, and decreased hospital readmission for infants receiving FCC interventions compared to those receiving usual care. However, parent involvement was not significantly associated with infant LOS. A narrative synthesis demonstrated additional benefits of FCC interventions for growth, short-term neurodevelopment, and parental well-being.

Considering the increase in neonatal FCC research in recent years and the ongoing diversity in interventions and outcome measures, there is a continuing need for the critical evaluation of emerging evidence. Therefore, this review focuses on summarizing and synthesizing the most recent research on FCC interventions and interpreting these findings in the context of the extensive analyses of prior studies.

### Objective

The purpose of this systematic review was to synthesize the research from the past five years that measured the impact and experiences of FCC interventions on infants and caregivers (parents). Two FCC models of care, FICare and Close Collaboration with Parents, were excluded from the review because they have been the subject of prior systematic reviews [3,11].

## 2. Materials and Methods

### 2.1. Study Design

We conducted a systematic review using the PRISMA guidelines [22]. See Supplementary Table S1 for the PRISMA checklist. We chose to include quantitative, qualitative, and mixed-methods research to provide a comprehensive review of neonatal FCC research, which is a complex phenomenon [23].

### 2.2. Search Strategy

We searched the databases PubMed, CINAHL, Embase, PsycInfo, and Web of Science for research published on or after 1 January 2017. We later limited the search to five years by excluding studies published prior to 1 September 2019. See Supplementary Table S2 for the search strategies for each database. We completed the search on 14 February 2023 and updated the search on 6 February 2024 and again on 1 September 2024. See Table 1 for the details of the inclusion and exclusion criteria. We imported the title/abstract results from the database searches into the Covidence software, Melbourne, Australia, [24] to identify and remove duplicates.

### 2.3. Selection of Studies

**Figure 1:**
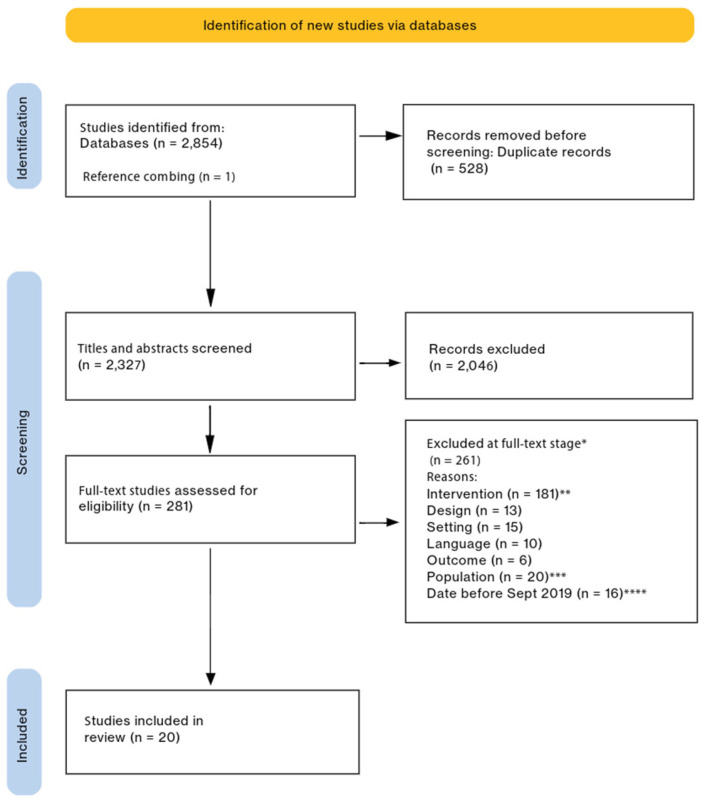
Preferred Reporting Items for Systematic Reviews and Meta-Analyses diagram [22]. * Full-text studies were allowed multiple reasons for exclusion based on a hierarchy of exclusion codes. Only the highest-ranked exclusion reasons for each study are reported here. ** Studies with a FICare intervention (n = 31) or with a Close Collaboration with Parents intervention (n = 3) were included in the initial search strategy but later excluded from this systematic review to avoid redundancy with other recent reviews. *** Studies with pediatric (non-NICU) patients were included in the original search but analyzed in a separate systematic review. **** We limited our review to the past five years to avoid redundancy with other recent reviews.

Using the inclusion and exclusion criteria, two study authors independently reviewed all titles and abstracts for the initial search results. If there was disagreement, the third author was consulted until a consensus was reached. We used the same process for the full-text stage of study selection, where the third author was a tiebreaker for 41 full-text articles. Studies were excluded if they did not meet the inclusion criteria or met the exclusion criteria. Our initial search included neonatal and pediatric inpatient settings; however, early in the systematic review process, we determined that each body of literature (pediatric and neonatal) warranted a separate review [25]. A systematic review of 15 pediatric inpatient FCC interventions was published previously [26]. In this neonatal-focused systematic review, the most common exclusion reason was the lack of an FCC intervention or approach. We excluded studies that only measured one aspect of FCC, such as SSC. Other decisions were made at subsequent stages of our review based on the relevance of the literature and the goal of a concise and organized synthesis. We chose to eliminate studies in NICU settings that implemented one of the standardized FCC interventions that have been recently synthesized and have a strong evidence base [1,3,11,12]. Thirty-one FICare intervention studies and three Close Collaboration with Parents intervention studies were excluded. See the PRISMA diagram in Figure 1.

### 2.4. Data Extraction Process

The first author independently extracted data from all 20 studies using a data extraction tool that we developed to organize and categorize the data from each study. The data extracted were as follows: title, abstract, year, population, aim, conceptual framework, design, start and end date, inclusion criteria, exclusion criteria, reason for not participating, recruitment method, FCC intervention, comparison group, baseline differences, withdrawals, all outcomes, confounders, data collection methods, quantitative instruments’ validity and reliability, statistical analysis, type of qualitative analysis, efforts to address bias, number of infant and parent participants, age of parents, age and GA of infants, sex of infant, parent role, parent race, parent age, all results, qualitative findings, effect size, sensitivity analysis, conclusion of study authors, limitations, and other notes. The second author extracted data from 25% (n = 4) of the studies and we compared the results. We discussed any differences until we reached a consensus. The third author was not needed at this stage. We focused our extraction on data relevant to infant and parent outcomes or experiences related to an FCC intervention in the NICU. Therefore, we did not extract irrelevant data in studies with multiple aims or study components such as staff interviews or health service outcomes. We extracted all primary and secondary outcomes for infants and parents from the 20 studies of 19 interventions.

### 2.5. Quality Appraisal

We critically appraised the quality of each article using a modified mixed-methods assessment tool (MMAT), an Excel spreadsheet tool that was established to evaluate quantitative, qualitative, and mixed-methods studies [27]. Our study team amended the MMAT to include detailed criteria for more precise grading comparisons across studies, resulting in quality percentage scores for each study. Two authors appraised the studies independently, and differences were discussed until we reached a consensus.

### 2.6. Synthesis

We used narrative synthesis [28] to present our analysis based on the a priori framework of FCC, including the core principles of respect and dignity, information sharing, participation, and collaboration [29]. We were not able to conduct a meta-analysis due to the heterogeneity of the outcomes and measurements. We summarized and organized the studies by the primary focus of each study’s FCC intervention. We evaluated mixed-methods studies by summarizing the results from the quantitative strand and qualitative strand and overall mixed-methods results. We synthesized all primary and secondary reported outcomes. When available from the studies, we noted the effect sizes in the narrative synthesis and show them in Supplementary Table S3. We reported the significant findings at *p* < 0.05 or smaller. We synthesized the qualitative results into themes and integrated the qualitative findings with the quantitative and mixed-methods findings into an overall narrative summary.

## 3. Results

### 3.1. Study Characteristics

See Supplementary Table S3 for a summary of the study characteristics and findings.

We found 20 studies that evaluated 19 FCC interventions in NICUs globally. Two studies reported different outcomes with the same intervention and sample. Sixteen studies were based on a quantitative design, including four cross-sectional studies [30,31,32,33], 10 quasi-experimental studies [34,35,36,37,38,39,40,41,42,43], and two RCTs [44,45]. Three studies used a qualitative design [46,47,48] and one used a mixed-methods design with a cross-sectional strand [49]. Four studies were quality improvement (QI) or program evaluation projects [30,36,42,49], three studies were pilot or feasibility studies [33,41,45,48], and two were retrospective analyses [40], including one that utilized EHR data [35].

The 20 studies included a total of 3502 parents/primary caregivers of infants in NICUs (range 10–1277 per study). The number of infants was not reported in every study, and most studies involved infant–parent dyads. Six studies included only mothers [38,41,42,45,46,47], one study included only fathers [48], and the remainder included both mothers and fathers. All studies took place in a neonatal setting, ranging from low to high acuity, although, in some studies, the NICU level of care was not specified. Fifteen studies took place at a single site [30,32,33,35,36,37,38,39,40,42,43,44,45,48,49], four in two different NICUs [34,41,46,47], and one included 66 NICUs [31]. The 20 studies represented 11 countries. Four studies were from India [33,41,42,44], four from Iran [34,38,39,40], three from the US [45,47,49], two from Germany [31,32], one from Finland and the US [46], and one from each of the following: Canada [30], China [43], Denmark [48], France [35], Norway [36], and Sweden [37]. All studies were conducted in NICUs. Most studies included only preterm or low/very low-birth-weight infants [31,35,37,38,39,40,41,44,47,48]. Other specific groups within the NICU setting were infants up to one year of age [45], medically complex infants [49], and infants in end-of-life (EOL) care [43]. The samples from other NICU studies were not specified.

### 3.2. Quality Appraisal of Studies [31,35,37,38,39,40,41,44,47,48]

The average quality score for the quantitative studies (including one mixed-methods quantitative strand) was 71%, with scores ranging from 38% [30] to 100% [35,48]. See Supplementary Table S4 for the MMAT quality appraisal scores. Overall, in terms of quality, the two RCTs were strong in assigning randomization and having complete data. One RCT [44] did not have comparable groups at baseline, and the other [45] did not have participants who successfully completed the FCC intervention. Neither RCT study had assessors who were blinded to the intervention. The ten quasi-experimental studies [34,35,36,37,38,39,40,41,42,43] were strong in reporting the inclusion and exclusion criteria, using appropriate measurements, collecting confounder data, and administering the FCC interventions as intended. The quasi-experimental studies’ weaknesses were not reporting why some parents did not participate in the intervention, not attempting to achieve a sample population that was representative of the target population, and not analyzing confounders. The five cross-sectional studies (including the mixed-methods strand) were strong in sampling a representative population and their sampling procedures, but only one study justified the sampling framework. All five cross-sectional studies generally had appropriate measures and statistical analyses. None of the cross-sectional studies reported pre-testing their surveys or described how they handled nonresponses from participants. While Table S4 provides the quality score for each study, we note, in the narrative synthesis, the strengths and weaknesses so that the reader may interpret the findings accordingly.

We scored the four qualitative studies (including one mixed-methods qualitative strand) between 67% [46] and 100% [48,49], with an average of 88%. All but one study used an appropriate qualitative approach and all but one used adequate data collection methods. All studies reported complete data, adequately derived the findings from the data, and substantiated their interpretations with quotations. All but one study had coherence throughout the research question, data sources, data collection, analysis, and interpretation. The one mixed-methods study [49] received a 50% score because the MMAT uses the lowest-scoring component (qualitative, quantitative, mixed-methods scores) as the overall score.

### 3.3. Outcome Measures

Half of the quantitative studies evaluated infant outcomes, including feeding, weight, infections, morbidity and mortality, painful procedures, medical errors, and LOS. The outcomes were described and measured differently across the studies. All quantitative studies measured at least one parent outcome, but the description of the outcomes and the instruments used to measure them were almost as diverse as the studies themselves. An exception was the similar use of a parent stress scale, the PSS:NICU, in three different studies. Mirlashari et al. [38] and Rajabzadeh et al. (2020) [39] both used validated Persian versions of the PSS:NICU [50] and reported total stress scores, while Månsson et al. [37] used a validated Swedish version of the PSS:NICU [51] and reported item-level mean score increases from before to after an FCC intervention. See Table 2 for the outcomes and measurements/qualitative methods from all 20 studies.

### 3.4. Study Findings by FCC Principle and Intervention Type

Since FCC is a multifaceted model of care in NICUs, many FCC interventions address more than one of the four FCC principles: respect and dignity, information sharing, parent participation, and collaboration. We presented the studies using the most closely aligned principles based on the description of each intervention. Eight interventions focused on information sharing, six on promoting parent participation in direct caregiving, and four on multiple principles, as described below. The FCC principle of collaboration was not included as a major principle for organization but rather evaluated in studies where it was reported that parents and healthcare providers collaborated at the organizational level. We found two studies where parents collaborated with the NICU research team, using pre-implementation surveys [49] and interviews [30]. Another study’s [43] collaboration involved previous NICU parent representatives in the creation of the study.

#### 3.4.1. Information-Sharing Interventions [34,37,38,39,40,41,45,48]

Eight studies evaluated seven FCC interventions focused on information sharing, including education sessions or modules [34,38,39,40,41], virtual family-centered rounds (FCR) [45], dialogs and daily information exchange [37], and knowledge-sharing groups [48].

##### Educational Interventions

Five studies that used quasi-experimental designs to evaluate educational FCC interventions will be discussed here. A range of educational topics and specific details about each intervention can be found in Table S3. For summary, here, we list whether the intervention contained general caregiving information, such as holding, bathing, diaper changes, umbilical cord care, sleep, safety, neurodevelopmental information, and infant conditions such as prematurity or jaundice. We considered feeding to include any of the following: breastfeeding, giving breastmilk or formula, orogastric tube feeding, and spoon feeding. Kangaroo care (SSC) and communication were other common topics.

Mirlashari et al. [38] examined the stress and coping levels of NICU mothers in Iran before and after an FCC educational intervention. Eighty mothers of infants less than 35 weeks GA were split evenly into intervention and control groups, and the intervention group received four group discussions covering general caregiving, feeding, and SSC. The results showed that the intervention group’s mean total stress scores decreased from pre- to post-intervention by 36%, compared to 8% in the control group; the mean problem-focused coping score (more desirable) increased by 5%, compared to less than 1%; and the mean emotion-focused coping scores (less desirable) decreased by 4%, compared to 1%. Unadjusted results were reported, but the potential confounders were comparable between the groups.

Two studies evaluated the same FCC educational intervention for parents of infants 30–37 weeks GA in a NICU in Iran [39,40]. The FCC educational intervention for both studies consisted of five daily sessions, with topics including general caregiving, feeding, SSC, communication, and psychological support. Rajabzadeh et al. (2020) [39] included 80 mothers, fathers, and infants divided equally into intervention and control groups and measured parents’ stress. The mean total scores on the PSS:NICU for mothers in the intervention group decreased from pre- to post-intervention by 42%, compared to 13% in the control group. For fathers, the mean decrease was 31% in the intervention group, compared to 10% in the control group. In the second study by Rajabzadeh et al. (2024) [40], the mean post-traumatic stress disorder (PTSD) scores were compared for a subsample of 40 mothers in the intervention group and 40 in the control group, from the same cohort as in the prior study. The mean total PTSD scores for mothers in the intervention group decreased from pre- to post-intervention by 20%, compared to 12% in the control group. Unadjusted results were reported for both studies on this educational intervention, as the potential confounders were comparable between the groups.

A pilot study in two NICUs in India enrolled 60 mother–infant dyads, split evenly into intervention and control groups, in an educational intervention with one-to-one teaching about general caregiving, feeding, SSC, communication, and psychological training [41]. These infants, between 30 and 34 weeks GA, and their mothers were observed during hospitalization on days 1, 7, and 14. The results showed that the intervention group of infants had higher mean increases in their PIBBS feeding scores (but not behavior changes or weight gain) pre- to post-intervention than the usual care control group. The mothers in the intervention group’s mean scores were significantly higher than those of the control group in all six competencies: communication and safety, feeding, positioning and SSC, prevention of infection, and skin care. However, the magnitude changes were not reported for any outcomes, and the analyses did not adjust for confounders, despite differences between the two groups.

A study by Khanjari et al. [34] enrolled 52 pairs of mothers/fathers and their 52 infants in two NICUs in Iran in a study with a pre- and post-intervention design. The parents received three FCC educational sessions that were 60 min each and included general caregiving and feeding. The fathers’ mean total QOL score and mean score on four of five components increased by between 11 and 17% from pre- to post-intervention. The mothers’ mean total QOL score and mean scores on all five components increased by between 12 and 22% from pre- to post-intervention. Mothers had higher mean increases than fathers. Fathers’ mean scores for the physical component of QOL decreased by 3%. Despite reporting unadjusted results, it was not reported that the potential confounders were comparable between the groups.

To summarize the infant outcomes of educational FCC interventions, no outcomes worsened, and there were improvements in feeding but not behavior or weight gain compared to the control group. Parent outcomes included one small negative impact on one of five QOL components. All other parent outcomes were positive, with improved stress, coping, competencies, and PTSD compared to the control group. One study reported improved QOL for mothers and fathers from pre- to post-intervention, without a control group.

##### Family-Centered Rounds Intervention

Rosenthal et al. [45] conducted a pilot RCT of a virtual FCR intervention in a US NICU for parents of infants at less than 365 days. Seventy-four mother–infant dyads were randomized to participate virtually in daily rounds via Zoom, and 36 received usual care, where parents had the option to join in-person rounds only. Of the five primary feasibility objectives, three were met, namely technical issues, time burden, and data collection, and two were not, namely recruitment and intervention uptake. Infants in the intervention group had mean increased exclusive breastmilk feeding rates at discharge of 31%, compared to 11% in the control group, and a shorter median LOS by eight days. Parent outcomes included higher composite caregiver experience mean scores in the intervention group of 65.4%, compared to 21.5% in the control group. Unadjusted results were reported as the potential confounders were comparable between the intervention and control groups.

##### Dialogs and Daily Information Exchange Intervention

One quasi-experimental study conducted in a NICU in Sweden with preterm infants less than 37 weeks GA and their parents compared parents’ experiences of different groups before and after an FCC intervention [37]. In the control group (before the intervention), there were 49 fathers, 49 mothers, and 64 infants. The intervention group had 60 fathers, 58 mothers, and 58 infants. The neonatal support program intervention included four dialogs and daily information exchange between parents and professionals. There were no differences in the mothers’ or fathers’ mean total or mean subscale scores on the PSS:NICU between the control and intervention groups. The analysis did not adjust for potential confounders or multiple comparisons.

##### Knowledge-Sharing Group Intervention

A qualitative descriptive study in a NICU in Denmark involved ten fathers and 14 infants (two sets of twins) between 26 + 1 and 36 + 5 GA [48]. Fathers who participated in semi-structured interviews had attended one or more knowledge-sharing groups in the NICU. The sessions were led by a neonatologist who was also a father and focused on general caregiving and peer support. The overall theme derived from the analysis was “emotional support, encouragement, and an enhanced capacity to deal with the situation and life in the NICU”. This theme encompassed how the groups helped fathers to increase their self-efficacy and confidence by sharing their experiences with their peers.

#### 3.4.2. Interventions to Promote Parent Participation in Direct Caregiving [30,33,35,36,43,47]

Six studies evaluated an FCC intervention that focused on promoting parents’ participation in their infants’ hospital care.

Klein et al. [35] conducted a quasi-experimental study of a parent participation intervention in a NICU in France with the parents of extremely preterm infants (less than 28 weeks GA). The electronic health records (EHRs) of the infants were examined at three time points: before the intervention, 2007–2008, 54 infants; during the intervention, 2010 to 2011, 77 infants; and after the intervention, 2013 to 2014, 97 infants. The Neonatal Individualized Developmental Care and Assessment Program (NIDCAP) is a complex FCC intervention based on neurodevelopment, parent–infant interactions, parent involvement, and breastfeeding promotion. Comparisons between the third cohort (intervention group) and first cohort (control group) found that infants had fewer painful procedures, with a mean of 37 compared to 50, and more pain evaluations, with a mean of 46 compared to three. Infants weighed an average of 359 g more at discharge. Infants receiving at least one SSC session increased by a mean of 67%; the first SSC was performed, on average, four days earlier and lasted twice as long; and the duration of infants’ mechanical ventilation decreased by an unspecified amount. Parents’ presence with their infant increased, with a mean of 40 h per week compared to 26. There were no differences in other infant outcomes. The analyses were adjusted for the gestational age.

Another quasi-experimental QI project in a NICU in Norway compared 37 infant–parent dyads before a participation intervention to a different group of 52 infant–parent dyads after the intervention [36]. The FCC cultural change intervention was a COMFORTneo pain training program for staff, with the goals of facilitating parent participation and parent presence for painful procedures, improving pain management guidelines, and providing ergonomic equipment to facilitate parental involvement. Only descriptive results were reported, and they showed an increase in overall parent participation by 32% from the control to intervention groups. The increases in parents’ participation by procedure were 22% for venipuncture, 23% for the insertion of an NG tube, 70% for the insertion of PVC, and 25% for airway suction.

A quasi-experimental study in a NICU in China included 45 infants receiving EOL palliative care and their parents [43]. Allocation to a family-supportive EOL care intervention group or routine control group was by the parents’ choice. This resulted in 20 infant–parent dyads in the intervention group and 25 dyads in the usual care control group. The intervention included single-bed rooms, parents participating in basic care, physical contact with their infant, creating commemorative items, and meeting with a psychologist every day. All parents completed a depression scale one week after their infant’s death. For the intervention group of mothers, the mean score on the EPDS depression scale was 2% lower than in the control group. Fathers in the intervention group had mean scores on the HAMD depression scale that were 3% lower than in the control group. All parents in the intervention groups reported satisfaction scores that were 2% higher than in the control group. This study was one of the three studies that reported collaboration in study development, specifically by including previous NICU parent representatives in the study design team. However, these differences were small in magnitude, and the analyses were not adjusted for confounders despite differences between the two groups.

A cross-sectional feasibility study in a NICU in India assessed an FCC intervention with a sample of 124 father–infant dyads and 320 mother–infant dyads [33]. The FCC program was a paradigm shift in this NICU, considering parents as willful participants instead of passive observers. An FCC comprehensive audio–visual tool had four sequential training modules about typical caregiving and feeding. Descriptive results were reported, including the fact that 76% of the planned parent training sessions were held, 50% of parents completed all four training sessions, 95% completed the first two sessions, 60% completed session three, and 75% completed session four. From the first to the second month of the intervention, the percentage of parents meeting some competencies increased, including maintaining personal hygiene by 29%, the handwashing duration by 17%, cleaning the baby properly by 6%, breastfeeding positioning by 2%, and the milk expression technique by 8%. Other competencies decreased, including handwashing before entering the NICU by 22%, handwashing steps by 8%, and the positioning of the infant by 26%. There were no differences in the removal of accessories or removal of footwear, which were 0% at both time points.

Another cross-sectional implementation and evaluation program was completed in a NICU in Canada during the COVID-19 pandemic [30]. The first part of the FCC intervention was an audio program, facilitated by a music therapist, where nurses played recordings of parents speaking, singing, or reading to their babies in the infants’ incubators. The second part of the intervention was a program of video chats between parents and babies. The sample sizes for the audio and video program evaluations were small, with 16 and 6 parents, respectively. For the audio program, the staff reported improvements in infant calmness with their parent’s voices, at 85%. Parents reported feeling less stressed, at 93.8%, and a strengthened bond with their baby, at 87.5%, while none reported feeling uncomfortable or embarrassed, at 0%. The video program resulted in parents feeling more involved in their infant’s care, at 100%; reduced stress, at 100%; and a stronger bond to their baby, at 66.7%, and to the NICU team, at 83.3%. The parents did not feel that the video program caused them to visit the NICU less frequently, at 0%, whereas one parent, amounting to 6.3%, in the audio program reported visiting the NICU less frequently since participating in the intervention. Two parents, or 33.3%, reported technical difficulties with the video technology. A strength of this study was the parent and healthcare team collaboration, as evidenced by the intervention development based on parent survey responses.

A qualitative study based in two US NICUs explored the experiences of mothers whose infants were born before 32 weeks GA [47]. A purposive sample of 14 mothers was interviewed about their experiences of visiting, general caregiving, holding, and feeding their infant (a priori themes developed from a literature review of mothers’ experiences two decades ago). Both NICUs had implemented FCC interventions with slight differences. The results included the following themes: (1) visiting—barriers included birth complications, childcare, finances, and a comfortable place to sleep; (2) general caregiving—mothers expressed anxiety, high levels of participation in care, knowledge, growing confidence, and positive relationships with nurses; (3) holding—began between their infants’ days of life 3–10 for the group, and most mothers valued SSC and began managing their own holding over time; (4) feeding—half of the mothers gave their infants breastmilk and reported some challenges, but they had good lactation support; (5) maternal ideas for improvement—specific NICU environment suggestions, more support groups, and more check-ins from staff about mothers’ needs for resources.

To summarize the infant outcomes from interventions to promote parent participation, there were no worsened outcomes, and two studies had improved infant outcomes. One large study found a decreased number of painful procedures, increased pain evaluations, a higher mean weight at discharge by 359 g, substantial increases in SSC and parent presence, and decreased mechanical ventilation use compared to a control group [35]. A different small study found that 85% of infants appeared to calm during the intervention, as reported by their nurses. The parent outcomes for this group of participation intervention studies included increased presence and participation and decreased depression scores by 2–3% compared to the control groups. Two descriptive studies had mixed findings with mostly positive outcomes, but one parent visited their infant less due to the intervention and two experienced technical difficulties in one study. The other study measured parent competencies and found that five improved, three worsened, and two did not change. One qualitative study provided in-depth knowledge about mothers’ experiences with caregiving, feeding, and SSC.

#### 3.4.3. Respect and Dignity Interventions [49] 

One study reported an FCC intervention focused on the principles of respect and dignity [49]. This mixed-methods study in a US NICU involved interviewing five parents and surveying 23 parents of infants with medical complexities to evaluate an FCC longitudinal care program. Families with higher social needs were prioritized for the program. Each family in the intervention was paired with an inpatient coordinator, followed by an outpatient coordinator for a year after discharge. Most parents, at 87%, reported meeting with their child’s inpatient care team, and 73% of parents met with their outpatient coordinator more than once per week. One parent reported difficulty in contacting their care coordinator for specific questions. Ninety-six percent of parents felt comfortable advocating for their child’s needs in the NICU. The parents’ mean score for overall satisfaction was 1.2 on a scale of 1–4, with lower scores indicating higher satisfaction. Only one parent was dissatisfied. Four general themes summarizing parents’ experience were (1) logistics, (2) communication, (3) financial support, and (4) emotional support. Despite its low quality score, this study stood out because it was the only study to report subgroup analyses by race (see Table S3). This study was also one of the few that included the FCC principle of collaboration by involving parents in the intervention development process.

#### 3.4.4. Multiple-Principle FCC Interventions [31,32,42,44]

##### Studies That Focused on Both Information Sharing and Parent Participation in Direct Caregiving

One of the two RCTs in our review was in a NICU in India, where the parents of infants born between 28 and 32 weeks GA participated in a two-group study design to evaluate an FCC intervention [44]. Seventy-three infants were randomly assigned to the FCC group, while 74 received routine care. The FCC intervention was called the Early Parent Participation Program (EPPP) and started on the first or second day of life. Five education modules were provided, including general caregiving, feeding, and SSC. Significant results were decreased infant physiologic instability, from 66% in control group infants to 47% in intervention group infants; decreased infant feeding intolerance, from 36% to 18%; and increased parent confidence in performing skills, from 73% to 89%. Non-significant improvements were seen in infant apnea, sepsis workups, breastfeeding rates at discharge, and early discharge. There were no differences in many infant mortality and morbidity outcomes between the groups. These findings were not adjusted for confounders despite differences between the two groups.

A quasi-experimental quality improvement (QI) project in a NICU in India compared different groups at baseline and during and after an FCC intervention [42]. Ninety-eight parent–infant dyads were in the baseline group, 258 dyads were in the intervention group, and 149 dyads were in the post-intervention group. The FCC intervention included expanded visitation hours, parental education through audio–visual aids, and capacity building through training and peer support. Hands-on training was provided to mothers about general caregiving and feeding. Statistical process control results were reported, and the increased participation of mothers from baseline to intervention to post-intervention was 32%, 44%, and 66%. There were no differences in the incidence of sepsis rates from before to after the FCC intervention.

The largest study in our summary was a cross-sectional design with multi-level regression modeling to determine the predictors of parent satisfaction across 66 NICUs in Germany [31]. A sample of 1277 parents of 923 infants reported their degree of satisfaction, and their scores were compared with the components of existing FCC practices, including recreation rooms, rooming in, unrestricted visiting hours for parents, parent classes, connection to parent associations, and standards on developmentally supportive care. In the adjusted analyses, two of the six variables were significant predictors of parent satisfaction: unrestricted visiting hours and standardized procedures for developmentally supportive care.

A cross-sectional study of 67 infants in a NICU in Germany explored the rates of viral respiratory tract infection (VRTI) during an existing FCC intervention, which included education about general caregiving, SSC, communication, and peer-to-peer support [32]. The NICU did not have permanent rooming in, but parents had a lounge area and a separate family home for overnight stays. The NICU was open 24/7 to families and close friends, two at a time, and for siblings with a health screening. Infection control measures were implemented. Measurements included a total of 75 symptomatic screenings and 272 scheduled weekly screenings. Descriptive results were reported. Two infants (3%) developed a VRTI, and no new VRTI cases were diagnosed during asymptomatic screenings. The study established a low VRTI rate despite the high rate of family visits.

##### Studies That Focused on Information Sharing, Parent Participation in Direct Caregiving, and Respect and Dignity [46]

A qualitative grounded theory study explored parent experiences in two NICUs, in the US and Finland, where FCC interventions were implemented [46]. Families of preterm infants less than 32 weeks GA or VLBW, whose mothers decided to breastfeed or pump milk, were included. Both units had established FCC, including FCRs, but took a different philosophical approach to the parents’ inclusion in medical decision-making. Parent participation in infant care was more encouraged in the Finnish than in the US unit, although both supported early SSC. Most of the US NICUs had open-bay architectures, while the Finnish NICUs had mostly single-family rooms. Using the same interview techniques, the researchers interviewed seven mothers in the US and eight mothers in Finland about their lived experiences related to feeding. The results included a global theme of lactation as a means or an end, which showed that lactation and infant feeding were framed differently in each location. Supporting themes that explained families’ perceptions of their transition to parenthood, family unit support, and lactation experiences included universal early postnatal challenges, culture- and space-dependent nursing support, and controlled or empowering breastfeeding experiences.

In summary, the interventions that focused on multiple FCC principles had no worsened outcomes. There were improvements in infants’ physiologic stability and feeding tolerance compared to the control group, and, in another study, there was a low incidence of viral respiratory infection despite a high rate of family visits. Parent outcomes were improved confidence and participation compared to control groups and the finding that visiting hours and standardized procedures for developmentally supportive care were predictors of parent satisfaction. No parent outcomes worsened. A qualitative study reported rich data from interviews with mothers in NICUs in the US and Finland and compared their experiences in breastfeeding.

### 3.5. Synthesis of Findings

#### 3.5.1. Quantitative Synthesis

##### Synthesis of Infant Findings

This review included eight studies that measured infant outcomes, including feeding, weight, LOS, behavior, infection, mortality, and morbidities. Four studies [35,41,42,44] evaluated infant feeding outcomes and three found significant improvements between the intervention and control groups. All feeding measures were different but improved for information-sharing interventions [41,45] and a participation intervention [44]. No studies reported worsened feeding outcomes. Three studies measured the infant weight [35,41,44], and one found an increase in the mean weight at discharge after a parent participation intervention [35]. No studies reported weight loss. Three studies measured the infant LOS [35,44,45], and one found an 8-day-shorter LOS between infants in the information-sharing (FCR) group and infants in the control group [45]. No studies reported a longer LOS for an intervention group. In summary, regarding the infant findings, we found the most evidence for improved outcomes in feeding and less evidence for the outcomes of infant weight gain and LOS. No other infant outcomes were significant, and no infant outcomes worsened.

##### Synthesis of Parent Findings

Four of seven studies [33,35,36,42,44,45,49] that measured outcomes related to parents’ participation in care had significant mean/median differences compared to the control groups, including earlier participation by six days [44], three times higher parent attendance [45], weekly increased presence by 14 h of presence and caregiving by eight [35], and improved care competencies [41]. Two studies reported descriptive improvements in participation [36,42], while one study had mixed results in terms of participation [33]. No participation measures worsened in any study.

Four of six studies [30,31,43,44,45,49] that measured parents’ satisfaction with FCC interventions reported significant differences compared to the control group, including increased measures of satisfaction of 44% [45], 16% [44], and 3% [43], and the finding that unrestricted visiting hours and standardized procedures for developmentally supportive care were predictors of satisfaction [31]. Only a small number of parent participants across all studies reported dissatisfaction.

Regarding parents’ mental health outcomes, two of three studies [37,38,39] that measured parents’ mean stress scores on the PSS:NICU reported significant increases compared to the control groups [38,39]. Additional findings across the studies were improvements compared to control groups in mothers’ coping measures [38], mothers’ and fathers’ depression [43], and mothers’ PTSD [40]. No study reported worsened mental health outcomes for parents.

The other significant differences in parent outcomes were the mean QOL scores in mothers and fathers, in which all five component QOL scores increased by 11–22%, with the fathers’ physical component decreased by 3%. In summary, regarding the parent outcomes related to FCC interventions, we found good evidence for improved participation, satisfaction, and mental health symptoms (stress, depression, PTSD, coping) and one study reported improved QOL outcomes. Very few parent outcome measures worsened, and each of these declined by a small magnitude. These collective findings came from FCC interventions of all types, including respect and dignity, information sharing, participation, and a combination of principles. We were not able to identify a type of FCC intervention that had superior efficacy.

#### 3.5.2. Qualitative and Mixed Methods Synthesis

Four qualitative studies/strands explored parents’ experiences of FCC interventions that included existing FCC care [46,47], fathers’ knowledge-sharing groups [48], and a longitudinal care coordination program [49]. We combined the resulting themes, and they aligned with the principles of FCC. The principles of respect and dignity was highlighted by two studies. Holdren et al. [46] explored the lived experiences of breastfeeding mothers in the US and Finland. The resulting themes showed that parents experienced lactation differently across cultures; mothers in the US NICU felt more controlled, while mothers in the Finnish NICU felt more empowered. Another study explored the experiences of the fathers of preterm infants after participation in a support group led by a neonatologist and found themes related to emotional support and a safe place to share feelings [48]. Themes that aligned with information sharing were discovered in a mixed-methods study where parents, who were paired with care coordinators, shared data that created themes about communication [49]. Themes that aligned with participation were found by Neu et al. [47], who explored the challenges and facilitators of parents’ participation in care, including visiting, general caregiving, holding, and feeding. These qualitative studies collectively underscored the importance of respect and dignity, information sharing, and participation in FCC interventions across cultures. The qualitative findings corroborated the quantitative findings and provided nuanced information about how and why the interventions worked for the parents who participated in the studies.

## 4. Discussion

### 4.1. Summary of Key Findings

This systematic review synthesized 20 studies from 11 countries that evaluated 19 FCC interventions and involved 3502 parents of infants in NICUs. These studies provide further evidence to support the effectiveness of a range of FCC interventions in the NICU, which have benefits for both infants and parents, and scant evidence of worsened outcomes or adverse incidents. The most common improved outcomes for infants were feeding and weight, consistent with prior systematic reviews [5,20,21]. Our review included one study with a finding of a decreased LOS. One of three previous reviews found a decreased LOS [21], while two reviews did not [5,20]. With regard to parent outcomes, the studies consistently showed improved parent participation, experiences, and satisfaction across a range of different FCC interventions and outcome measures, consistent with prior FCC reviews [5,20,21]. To a smaller degree, we found evidence for decreased parent stress, PTSD, and depression related to different FCC interventions, also consistent with prior FCC reviews [20,21]. The qualitative studies gave voice to parents’ lived experiences of participating in FCC interventions and provided context regarding how these outcomes are experienced and why FCC is so important to them, aligning with findings from previous qualitative research summaries evaluating parent participation and communication interventions [18,65]. Overall, however, there were only two new RCTs (one of which was a pilot RCT), and the quality of the studies varied widely, with an average score of 71% for quantitative studies, 88% for qualitative studies, and 50% for the mixed-methods study.

#### Comparison to the Systematic Review of FCC Interventions for Hospitalized Pediatric Patients

As detailed in Section 2, we conducted our search to learn about FCC interventions in hospitalized children aged preterm to 18 and subsequently split the review into two analyses. The earlier review [26] about FCC interventions in pediatric hospital settings found 16 studies on 15 FCC interventions in six countries, compared to 20 studies on 19 interventions across 11 countries in this review. The earlier review lacked RCTs (compared to the two in this review) but had similar representations of quasi-experimental, cross sectional, qualitative, and mixed-methods studies. The average quality score of the pediatric studies was 64%, compared to 71% in this review, with similar quality score ranges (36% to 100% versus 38% to 100%). The most common focus of the FCC interventions in both the pediatric and NICU reviews was information sharing (eight in each). However, the pediatric review had four interventions focused on respect and dignity, compared to two in this review, and two parent participation interventions, compared to six. The pediatric review had two mixed-principle interventions, compared to four in this review. The most striking difference between the two reviews was the number of studies reporting parent collaboration in designing and implementing an FCC intervention, which was nine out of 15 in the pediatric review and only two out of 19 in this review. The evidence base for pediatric FCC has historically lagged behind that of the NICU [4,15]. Therefore, it is surprising to find that collaboration between parents and researchers was more prevalent in the pediatric review than the NICU review.

The pediatric review found no significant differences in child outcomes after an FCC intervention, and few studies measured any child outcomes. In contrast, half of the quantitative studies in this review measured infant outcomes, and some found improvements in feeding and weight, while one study found a decreased LOS. The pediatric review summarized significant improvements in parent experience, satisfaction, knowledge, communication, self-efficacy, and spiritual well-being and decreased stress and anxiety after FCC interventions. The findings that overlap with this review are improved parent experiences and satisfaction and decreased stress and anxiety. This review additionally found improvements in depression and PTSD, most measures of QOL, and parents’ participation in their infants’ care. Participation in care was not a common outcome in the pediatric review. Both reviews found scant worsened outcomes.

### 4.2. Recommendations to Address Gaps and Unanswered Questions in NICU FCC Research

Despite the increase in recent studies about FCC interventions in NICUs globally, gaps persist in the quality of the sampling, study designs, methodologies, and measurements.

#### 4.2.1. Sampling

Studies in the NICU have traditionally included more mothers than fathers [66], yet over half of the studies in our review included both mothers and fathers, which suggests greater attention to parental gender in study recruitment. However, six studies focused exclusively on mothers, while only one focused exclusively on fathers. None of the studies in our review acknowledged lesbian, gay, bisexual, transgender, and queer (LGBTQ+) parents, who may be predisposed to negative health outcomes due to adverse experiences across the life course [67]. Only one study addressed diversity in racial/ethnic identities or socioeconomic status in the sampling strategy. Dallas et al. [49] prioritized recruiting families of higher social needs. Our findings are consistent with previous evidence of the underrepresentation of traditionally marginalized families in FCC research [68]. These gaps should serve as a call to action for future FCC research to develop sampling and recruitment strategies to increase the representation of traditionally marginalized populations and ensure equitable health outcomes. Community-based participatory research methods with active stakeholder engagement in study design and implementation are evidence-based approaches to improve research engagement and representativeness [69].

Our findings were consistent with the bibliometric research findings of Hriberšek et al. (2024) [16] indicating that the top ten countries publishing neonatal FCC literature since 2000 were Australia, Canada, China, Germany, Iran, Italy, the Netherlands, Sweden, the UK, and the US. We found research from similar global regions. Specifically, this review represented six of the top ten countries (Canada, China, Germany, Iran, Sweden, and the US) and Denmark, Finland, France, India, and Norway. Our synthesis echoes the need to include families in the development of interventions, as suggested by Hriberšek et al. [16].

#### 4.2.2. Study Design and Methods

The body of FCC research continues to lack an adequate number of RCTs. Only two studies in this review were RCTs, and eight were quasi-experimental designs. None of the studies compared an FCC intervention to another intervention but instead compared intervention groups to groups receiving usual care, often poorly defined. Furthermore, five studies compared different groups of parents from pre-intervention to post-intervention, which is a risk for selection bias. Our search criteria included studies that were designed as QI projects, program evaluations, pilot studies, or feasibility studies to capture as much knowledge as possible. However, we realize the limitations of these preliminary study designs. There is a need for well-designed, rigorous studies such as RCTs to evaluate the effectiveness of FCC interventions in the NICU. Cluster randomized trials and implementation science study designs are preferred to address the difficulties in masking FCC interventions, the variations in FCC, and the many unmeasured potential influences on NICU care delivery across sites [70,71]. Our review found that 15 studies took place in a single NICU, suggesting that more multi-site studies are needed. We also call for more longitudinal studies to measure outcomes that may not appear until well after discharge and long-term studies to evaluate child and family outcomes months or years after an intervention. Equally important is reporting the magnitude of the effects of FCC interventions so that we may better understand the clinical relevance of the findings.

Four high-quality studies in this review explored parents’ perceptions of FCC interventions. Parents’ narratives gave information about the broader context of their experiences when their child was in the NICU and uncovered the emotional and practical challenges of participating in care for their child. More qualitative research is needed, specifically focused on parents’ experiences of FCC interventions in the NICU setting. Qualitative research can give parents a voice regarding which outcomes are important to them and how FCC interventions work best for them. We need more evidence of the facilitators and barriers to parents’ participation in care, especially from parents of traditionally marginalized backgrounds. Knowledge about parents with diverse backgrounds is lacking but critically needed to gain a deeper understanding of how FCC interventions can reach and benefit every infant and family.

#### 4.2.3. Outcome Measurement

Like previous research [9,13,14], we found heterogeneous outcomes and measures related to FCC interventions in the NICU. The diversity of the measures made comparisons across studies difficult. We found no evidence published in the past five years indicating a movement toward a consensus on FCC measures. However, the PSS:NICU continues to be reported the most frequently as a measure of parental stress, as evidenced by three studies in our review [13]. Some FCC experts have called for a core set of outcome measures that can be created by consensus across NICUs for FCC research, including which outcomes and at which time points to evaluate FCC interventions. Additionally, an ideal core outcome set would include measures to report on the reliability and validity of the instruments [72,73]. Our findings support the need for core outcomes in NICU research about FCC interventions.

#### 4.2.4. FCC Interventions

The most common FCC interventions focused on information sharing, including educational programs, FCRs and other types of daily information exchange, and parent groups. Parent participation in their child’s care was the second most prioritized FCC principle. Four studies described multiple FCC principles [31,32,42,44], and we recommend this comprehensive approach for future FCC intervention research. The principle of respect and dignity was less well represented, with only two interventions focused on honoring patient and family perspectives and choices and incorporating families’ knowledge, beliefs, and cultural backgrounds. We encourage all future studies to include respect and dignity in developing and implementing FCC interventions. Finally, the principle of collaboration was underutilized in the studies that we reviewed. The parents of NICU infants should be encouraged and valued for participating in program and policy development, professional education and research, and delivering care to their infant.

### 4.3. Limitations of the Systematic Review

This systematic review reports on FCC NICU intervention research in the past five years, using the principles of FCC as an organizing framework. However, we may have missed important evidence for several reasons. First, we excluded 10 non-English studies from our original search. We had a broad initial search strategy, which included pediatric and neonatal settings. We decided that each research setting merited its own review, so we conducted two separate systematic reviews based on the original search [25,26]. While publishing the pediatric systematic review took time, we had to repeat the search for the NICU studies, which may have missed evidence. We made several decisions about our final selection of studies, all of which we reported transparently (see Figure 1), but this may have eliminated relevant evidence from the studies we excluded. When examining the 20 studies of 19 FCC interventions, we excluded findings related to healthcare staff or health service outcomes, which may have provided greater insights into FCC intervention uptake and environments. We were not able to conduct a meta-analysis due to the heterogeneity of the outcomes and measurements, but this would have strengthened our findings and allowed us to systematically assess publication bias.

### 4.4. Implications for Practice and Policy

The findings from the FCC intervention research in NICUs in the past five years, as well as previous reviews [5,20,21], suggest that NICUs have access to a range of promising FCC interventions. Multi-disciplinary teams, guided by collaboration with families, should be actively implementing and evaluating the quality and safety of FCC interventions.

Policymakers must prioritize FCC as the standard of care in all NICUs and integrate FCC interventions into practice. There is wide variation in FCC applicability across countries. For example, some countries still have restricted visiting hours for parents in the NICU, some have more comprehensive parental leave policies at workplaces, and some have larger inequities across populations in accessing healthcare. These discrepancies highlight the need to encourage more standardized approaches to FCC in NICUs globally.

### 4.5. Conclusions

With great respect for the researchers carrying out FCC intervention studies worldwide, we still find a lack of consistent, comparable studies. This is a problem that has characterized FCC research for decades. There have been recent advances in FCC science with specific programs such as FICare [1,3] and CCP [10], and we encourage NICU teams to consider these programs for their use. There is also great potential for novel interventions, especially when evaluated with rigorous methods and validated outcome measures. Finally, we recommend utilizing the FCC framework and its core principles to provide conceptual rigor for the development, implementation, and evaluation of interventions.

## Figures and Tables

**Table 1:** Inclusion and exclusion criteria.

	Inclusion Criteria	Exclusion Criteria
Study Design	Quantitative, including experimental (RCT, pre–post), observational (comparative, descriptive, retrospective); qualitative; mixed methods. Published in peer-reviewed journals.	Systematic review, other review, commentary, case study of only one, concept analysis, policy statements, opinion pieces. Studies from non-peer-reviewed journals.
Population	Children premature through age 18 and their parents/primary caregivers. We use the term “parent” to include mothers, fathers, or other primary caregivers.	Studies that did not include hospitalized children or their families. * Non-NICU samples were later excluded.
Setting	Hospitalized/inpatient	Studies in ambulatory or primary care settings, community settings. * Settings outside the NICU were later excluded.
Intervention	FCC interventions, programs, or models that include one or more components: physical or psychological support for parents, communication with parents, education, partnership, shared decision-making, or parent participation in care. Must promote core principles of FCC of respect and dignity, information sharing, participation, and collaboration.	Studies that do not describe or evaluate an FCC model, approach, or intervention. Studies that involve only one specific technique or only the physical environment. * Studies exclusively about SSC or kangaroo care were excluded. * Studies about FICare or CCP interventions were later excluded.
Comparison	Other models of care or interventions that do not explicitly include FCC interventions (for studies with a comparison group). Not applicable to qualitative research.	
Outcomes	Parent/family outcomes: knowledge or understanding, physical or psychological health, satisfaction, attitudes, behavior, interaction with child, adverse events for child or parent. Infant outcomes: physical, developmental, LOS.	Staff outcomes/experiences, health service outcomes.
Language	Published in English.	Any language other than English.
Publication Date	Between 1 January 2017 and 1 September 2024. * Later narrowed to between 1 September 2019 and 1 September 2024.	Prior to 1 January 2017. * Later narrowed to after 1 September 2019.

* Changes made after search was complete.

**Table 2:** Quantitative Outcomes and Measures/Qualitative Methods.

Author Year [Reference]	Outcome/ Measurement/ Qualitative Method	Developed for the Study/ Previously Validated or Both	Number of Items, Psychometric Properties, Measurement Development, Notes.
Antinora et al., 2023	Parents FCC Experience Surveys	Developed for the study	Pre-implementation parent survey, 20 items, Likert sale Post-intervention parent surveys for voice recording program, 12 items and video chat program, 13 items, Likert scales All surveys were completed on paper while in the NICU. Surveys had a section for additional comments. No reliability or validity reported
Dallas et al., 2022	Mixed methods Quantitative strand Parents Family experience and perception of care (FECC) survey Qualitative strand Parents Semi-structured interviews	Both Developed for the study and previously validated	FECC Overall experience, 4-, 5-, or 6-point Likert scales Inpatient experience, 23 items; outpatient experience, 22 items Validated by Parast et al. (2018) [52] Sent digitally to each parent Additional survey questions developed based on interview feedback. Published in appendix. No reliability/validity reported Interviews were 20 to 30 min via telephone or in person based on family preference Interview guide published in Supplementary Materials Focused on communication, parent role clarity, emotional support, knowledge and training, and financial resources
Holdren et al., 2019	Qualitative Parents Interviews	Developed for the study	Interview prompts included questions about becoming a NICU parent, infant feeding, provider interactions, and parenting post-discharge Interview guide not published Reported using the same interview methodology in the US and Finland until thematic saturation.
Jannes et al., 2020	Infants Weight BPD, ROP, infection, breastfeeding at discharge Parents satisfaction scale	Previously validated	EHR data Infant Change in weight z-scores from study days 1 to 22 Infant outcomes by incidence Parent satisfaction scale previously validated in German (Pfaff et al., 2004) [53] Current study’s confirmatory factor analysis, all items of the patient satisfaction scale were retained due to sufficient factor loadings Cronbach’s alpha, 0.87
Khanjari et al., 2022	Parents Quality of life (QOL)	Previously validated	QOLWHOQOL-BREF, 26-items, 5-point Likert scale Cronbach’s alpha from previous studies: physical health component, 0.77; social, 0.75; psychological, 0.77; environmental 0.84 (Nejat et al., 2005) [54] Cronbach’s alpha for present study: total, 0.86; physical health, 0.78; psychological 0.71, social function, 0.60, and environmental, 0.70
Kidzun et al., 2020	Infants Viral respiratory tract infections (VRTI)	Previously validated	Frequency of VRTI calculated by mPOCT of symptomatic infants combined with a weekly PCR screening of all infants Number of parents’ and siblings’ visits were recorded at the time of weekly screenings mPOCT and PCR, sensitivity/specificity not reported for either Virus samples (symptomatic) by nasopharyngeal swabs BioFireR FilmArrayR Respiratory RP2 Panel (mPOCT), which detects adenovirus, coronaviruses (HKU1, NL63, 229E, and OC43), human metapneumovirus, human rhinovirus/enterovirus, influenza (A, A/H1, A/H3, A/H1-2009, and B), parainfluenza virus 1–4 and respiratory syncytial virus Multiplexed PCR (weekly screening). 19-valent, in-house, multiplexed PCR method detects a similar range of viruses
Klein et al., 2021	Infants Painful procedures Pain evaluations Neurology and respiratory outcomes, LOS, NEC, infection, feeding, weight gain at discharge Parents Presence	Developed for the study	Frequency of painful procedures and pain evaluations documented by nurses EHR data All outcomes extracted from the medical records and nurses’ documentation Participation measured by: Presence with infant, in hours SSC, number and duration Participation in care, number of care procedures that included parents
Lægtskov et al., 2023	Qualitative Parents Semi-structured interviews	Developed for the study	Interviewed by phone or video call. Included field notes. Theme-based interview guide made after literature search and observation in the NICU, not published Initial interview question, “How was it for you to participate in the father group?” followed by specifying, interpreting, indirect, direct, and structured follow-up questions
Lyngstad et al., 2021	Parents Participation	Developed for the study	Nurses documented parent participation in painful and stressful procedures
Månsson et al., 2019	Parents Stress PSS:NICU	Previously validated	PSS:NICU, Swedish version. 35 items, 5-point scale, three subscales (infant behavior, appearance, and parental role alteration) and overall stress item Cronbach’s alpha from previous study, each subscale, 0.756–0.852 and homogeneity of the entire scale, 0.865 (Månsson et al., 2016) [51]
Maria et al., 2021	Parents Participation	Developed for the study	Nurses observed and recorded into study database. Compliance for each activity reported as percentages. For 10 activities: Personal hygiene, removing accessories, removal of footwear, handwashing, handwashing duration, handwashing steps, positioning infant, cleaning infant, positioning for breastfeeding, expressing milk
Mirlashari et al., 2021	Parents Stress PSS:NICU Coping The Brief COPE scale	Previously validated	PSS:NICU scale translated into Persian in previous studies Cronbach’s alpha, 0.87 (Beheshti-Pour et al., 2014) [50] Current study Cronbach’s alpha, 0.88 The BriefCOPE scale (Cheon, 2012) [55]. Translated into Persian previously (Nourali et al., 2013) [56]. Cronbach’s alpha for all subscales, 0.78 to 0.86 Current study Cronbach’s alpha, 0.72
Neu et al., 2020	Qualitative Semi-structured interviews	Developed for the study	Interviews in the NICU until thematic saturation Semi-structured, open-ended questions to explore mothers’ experiences Interview topics included pregnancy and birth experiences, visiting, social support, care, holding, feeding and discharge concerns; interview guide not published
Pillai et al., 2022	Infants Physiologic instability (PI) Secondary outcomes: breastfeeding at discharge, early discharge, mortality, sepsis, ROP, NEC, PVL, respiratory support, EUGR Parents Participation	Developed for the study	PI was defined as one of the following, (1) significant apnea, (2) feeding intolerance, or (3) sepsis workup. PI events recorded per infant and per study group Medical records Self-report sign-in sheet for parents
Rajabzadeh et al., 2020 and 2024	2020 Parents Stress PSS:NICU 2024 Retrospective Analysis Parents PTSD PTSD Checklist	Previously validated	PSS:NICU validated in Persian (Beheshti-Pour et al., 2014) [50] Current study Cronbach’s alpha, 0.88 Pre-test at admission, post-test at discharge PTSD checklist, 17-items, list of symptoms based closely on the DSM-IV criteria. Validated in Iran (Goodarzi et al., 2003) [57] Cronbach’s alpha, 0.93 (P = 0.0001, n = 117, r = 0.37) Current study Cronbach’s alpha, 0.88 Pre-test at admission, post-test at discharge
Rosenthal et al., 2021	Parents Attendance at FCRs Caregiver experience Child Hospital Consumer Assessment of Healthcare Providers and Systems (HCAHPS) Survey Infants Secondary outcomes: LOS, breast milk feeding at discharge, medical errors, adverse events	Previously validated	FCR was observed daily. FCR caregiver attendance proportion= number of weekday round encounters with at least one caregiver present (either virtually or in person) divided by the neonate’s total number of weekday round encounters Surveys, EHR data, incident reports and helpdesk technical issue logs provided data (HCAHPS) Survey (Toomey et al., 2015) [58], reliability and validity not reported. Top-box scoring of HCAHPS used (Giordano et al., 2010) [59] Survey was given at discharge EHR data
Saldanha and Gretta Tauro 2023	Infants Behaviors Preterm infant behavior scale Infant Feeding Preterm Infant Breastfeeding Behavior Scale (PIBBS) Infant physiologic parameters Parents Mothers’ competencies in infant care scale	Both Developed for the study and previously validated	Preterm infant behavior scale, (D’Souza et al., 2014) [60]. Reliability and validity not reported PIBBS (Nyqvist et al., 1999) [61]. Reliability and validity not reported Developed for the study: Competency of mothers in handling their neonates in the NICU, 4-point Likert scale, validated by 10 experts. Inter-rater reliability, Pearson correlation (r = 0.9) All measures were observed and recorded by the same nurse for both experimental and control groups
Sivanan-dan et al., 2021	Parents Participation Study balancing measures Sepsis Adverse events attributable to family participation	Developed for the study	Proportion of eligible mother-infant dyads participating in FCC (kangaroo Care, feeding, diaper changing). Daily documentation in a register. Total number of infants receiving care by family divided by total number of eligible infants that day Incidence of culture-positive sepsis cases per 100 NICU admissions during study period Adverse events tracked by statistical process control charts
Zhang et al., 2022	Parents Depression Edinburgh Postnatal Depression (EPDS) scale for mothers Hamilton depression rating scale (HAMD) for fathers (A different scale was used for depression because the EPDS was not validated in Chinese for men) Satisfaction	Both Developed for the study and previously validated	EPDS, Chinese version. 10 items, 4-point Likert scale, total of 30 possible (Maurer et al., 2018) [62] Cronbach’s alpha, 0.76 (Ijaz et al., 2018) [63] HAMD, Chinese version. 17 items, 3- or 5-point Likert scale, total of 78 possible (Nixon et al., 2020) [64] Cronbach’s alpha, 0.06 Depression scales administered to parents one week after an infant’s death Hospital standard satisfaction survey administered weekly to all patients by an external company 20 items, 5-point Likert scale, total of 100 possible. Topics included medical treatment, medical staff’s negotiation attitude, hospital setting, and social service

Notes: EHR, electronic health record; EUGR, extrauterine growth restriction; LOS, length of stay; mPOCT, multiplexed point-of-care testing; NEC, necrotizing enterocolitis; PCR, polymerase chain reaction; PSS:NICU, Parent Stressor Scale: Neonatal Intensive Care Unit; PVL, periventricular leukomalacia; ROP, retinopathy of prematurity; SSC, skin-to-skin care.

## Data Availability

The original contributions presented in the study are included in the article and Supplementary Materials; further inquiries can be directed to the corresponding author.

## Supplementary Materials

The following supporting information can be downloaded at: https://www.mdpi.com/article/10.3390/children12030290/s1, Table S1. PRISMA checklist (a) and abstract checklist (b); Table S2. List of Search terms; Table S3. Table of study characteristics, FCC interventions, and results; Table S4. Table of Modified MMAT Quality Assessment.
