# Supplementary material for: Infant and Family Outcomes and Experiences Related to Family-Centered Care Interventions in the NICU: A Systematic Review
Source: Children (Basel). 2025 Feb 26;12(3):290. doi: 10.3390/children12030290 (PMC11941216; doi:10.3390/children12030290)
Supplement: Supplementary file 1 [file children-12-00290-s001.zip › S2.pdf]

Supplementary Materials for Neonatal FCC Systematic Review

S1 PRISMA checklist and abstract checklist – see separate document
S2 Search terms
S3 Table of study characteristics, FCC interventions, and results
S4 Table of Modified MMAT Quality Assessment

S2 Database Search Terms

Family-centered care
Date searched: 2/13/2023
Dates search updated: 2/6/2024 and 9/11/24

PubMed:
("family-centered care" OR "Family Nursing"[Mesh] OR "family nursing" OR "family integrated care" OR "Patient-Centered Care"[Mesh] OR "patient-centered care" OR "patient-centered nursing") AND ("Child"[Mesh] OR child OR children OR "Adolescent"[Mesh] OR adolescent* OR teen* OR "Infant"[Mesh] OR infant* OR neonat* OR newborn OR pediatric) AND ("Inpatients"[Mesh] OR inpatient* OR hospitalized* OR "hospital-based" OR NICU OR "newborn intensive care" OR "Intensive Care Units, Neonatal"[Mesh] OR "pediatric ICU" OR "pediatric intensive care" OR "Intensive Care Units, Pediatric"[Mesh])
References = 1,178

Web of Science:
("family-centered care" OR "family nursing" OR "family integrated care" OR "patient-centered care" OR "patient-centered nursing") AND (child OR children OR adolescent* OR teen* OR infant* OR neonat* OR newborn OR pediatric) AND (inpatient* OR hospitalized* OR "hospital-based" OR NICU OR "newborn intensive care" OR "pediatric ICU" OR "pediatric intensive care")
References = 773

Embase (limit to articles/articles in press):
('family centered care'/exp OR 'family centered care' OR 'family integrated care'/exp OR 'family integrated care') AND ('pediatric'/exp OR 'pediatric' OR 'newborn'/exp OR 'newborn' OR 'infant'/exp OR 'infant' OR 'adolescent'/exp OR 'adolescent') AND ('hospital patient'/exp OR 'hospital patient' OR 'intensive care unit'/exp OR 'intensive care unit' OR hospitalized OR 'hospitalized child'/exp OR 'hospitalized child' OR 'hospitalized adolescent'/exp OR 'hospitalized adolescent' OR 'hospitalized infant'/exp OR 'hospitalized infant') AND ([article]/lim OR [article in press]/lim)
References = 477

CINAHL: (limit to scholarly articles)
("family-centered care" OR "family nursing" OR "family integrated care" OR "patient-centered care" OR "patient-centered nursing") AND (child OR children OR adolescent* OR teen* OR infant* OR neonat* OR newborn OR pediatric) AND (inpatient* OR hospitalized* OR "hospital-based" OR NICU OR "newborn intensive care" OR "pediatric ICU" OR "pediatric intensive care")
References = 1,507

PsycInfo:
("family-centered care" OR "family nursing" OR "family integrated care" OR "patient-centered care" OR "patient-centered nursing") AND (child OR children OR adolescent* OR teen* OR infant* OR neonat* OR newborn OR pediatric) AND (inpatient* OR hospitalized* OR "hospital-based" OR NICU OR "newborn intensive care" OR "pediatric ICU" OR "pediatric intensive care")

Table S3 Study Characteristics, FCC Interventions and Results

Author<br>Year<br><br>Country<br><br>[Reference]	Study Design<br><br>Quality Appraisal	Setting<br><br>Sample<br><br>Family Involvement	Intervention Description<br><br>FCC Principles (Primary Emphasis*)	RCT or Quasi-Experimental Results[1, 2]<br><br>Quantitative Descriptive Results<br><br>Qualitative Results
Antinora et al.<br>2023<br>[30]<br><br>Canada	Cross sectional<br><br>Implementation and evaluation project<br><br>Descriptive only<br><br>38%	Level 4 NICU during the COVID-19 pandemic<br><br>Parents<br>Audio program (n=48 families enrolled, n=16 included in analysis)<br><br>Video program (n=14 enrolled in video, n=6 included in analysis)<br><br>Intervention was developed based on parent and staff survey responses	Part 1: Audio program where recordings of parents' speaking, singing, or reading to their baby were played in the NICU infants' incubators when families could not be at the bedside. Nurses played the recording during care and procedures. Facilitated by a music therapist. Part 2: Video chats between parents and babies.<br><br>Respect and Dignity<br><br>Parent Participation*<br><br>Collaboration in Development	Descriptive Results<br><br>Improved<br>Audio Program<br>Infants<br>Became calmer with their parents' voices according to 85% of staff<br><br>Parents<br>Reported decreased stress, 94%,<br>Made them feel involved in their baby's care, 94%, Strengthened their bond with their baby, 88%, Made them feel uncomfortable or embarrassed, 0%<br><br>Video program<br>Parents<br>Felt more involved in their baby's care, 100%, Reduced their stress, 100%, Gave them a stronger bond to their baby 67%, and to the NICU team, 83%<br><br>No differences<br>Video Program<br>Make them visit the NICU less frequently, 0%

Author<br>Year<br><br>Country<br><br>[Reference]	Study Design<br><br>Quality Appraisal	Setting<br><br>Sample<br><br>Family Involvement	Intervention Description<br><br>FCC Principles<br>(Primary Emphasis*)	RCT or Quasi-Experimental Results[1, 2]<br><br>Quantitative Descriptive Results<br><br>Qualitative Results
				Worsened<br>Audio<br>Visited the NICU less since creating the recordings, 6%<br><br>Video<br>Had difficulty with the technology, 33%
Dallas et al.<br>2022<br>[49]<br><br>US	Mixed methods<br><br>Exploratory sequential with integration at the design level<br><br>Quantitative: Cross sectional<br><br>Qualitative: Interviews<br><br>Program evaluation<br><br>Quality scores<br>50% quant	Level 4 NICU in a freestanding academic children's hospital and at home through their first-year post-discharge<br><br>Parents of infants with medical complexity; families with higher social needs were prioritized<br><br>Parents<br>Interviews (n = 5)<br>Surveys (n = 23)<br><br>Parents were interviewed to elicit the family's experience and to evaluate the program	A novel longitudinal care coordination program with an inpatient coordinator helping navigate the healthcare system during their admission and hospitalization. Then, an outpatient coordinator helped with transition to home and stayed with each family for the first year after discharge.<br><br>Respect and Dignity*<br><br>Information Sharing<br><br>Parent Participation<br><br>Collaboration in Development	Descriptive Results<br><br>Parents<br>Frequency of communication<br>with outpatient coordinators, 73%<br>with inpatient coordinators, 83%<br><br>Overall satisfaction, mean score 1.2 (scale of 1-4 with lower scores indicating higher satisfaction). One parent was dissatisfied.<br><br>Parents' rating of the care coordinator's knowledge, 87%, Rating of the support for parent decisions, 91%<br><br>Significant differences in white vs minority/not reported families:<br><br>Length of enrollment, 23.7 vs 10.5 months

Author<br>Year<br><br>Country<br><br>[Reference]	Study Design<br><br>Quality Appraisal	Setting<br><br>Sample<br><br>Family Involvement	Intervention Description<br><br>FCC Principles<br>(Primary Emphasis*)	RCT or Quasi-Experimental Results[1, 2]<br><br>Quantitative Descriptive Results<br><br>Qualitative Results
	100% qual<br>50% mixed methods<br>50% overall<br><br>Descriptive and comparisons by race using Mann-Whitney *U* test and Fisher's exact test			Using help for reading hospital materials, 5 vs 4.1 (Likert scale with lower number meaning more help)<br><br>94% vs 60% reported participation in care<br><br>Four general themes summarizing the experience of parents were (1) logistics, (2) communication, (3) financial support, and (4) emotional support
Holdren et al.<br>2019<br>[46]<br><br>Finland and US	Qualitative<br><br>Grounded Theory<br><br>67%	US NICU, a regional tertiary care in a children's hospital<br><br>Finnish NICU, a regional tertiary care unit in a children's and women's hospital<br><br>Families of preterm infants <32 weeks GA or VLBW whose mothers decided to breastfeed or pump milk<br><br>US families (n=7)<br>Finland families (n=8)	Both units had established FCC including FCRs but had a different philosophical approach to the parents' inclusion in medical decision-making. Parent participation in infant care was more encouraged in the Finnish unit than in the US unit, although both supported early SSC. Most of the US NICU was open-bay architecture while compared to single-family rooms in Finnish NICU.	Evaluation of the lived experiences of FCC and feeding experiences<br><br>Qualitative Results<br><br>Global theme of lactation as a means or an end showed that lactation and infant feeding were framed differently in each location<br><br>Supporting themes that explained families' perceptions of their transition to parenthood, support as a family unit, and experience with lactation included: universal early postnatal challenges,

Author<br>Year<br><br>Country<br><br>[Reference]	Study Design<br><br>Quality Appraisal	Setting<br><br>Sample<br><br>Family Involvement	Intervention Description<br><br>FCC Principles<br>(Primary Emphasis*)	RCT or Quasi-Experimental Results[1, 2]<br><br>Quantitative Descriptive Results<br><br>Qualitative Results
			Respect and Dignity*<br><br>Information Sharing*<br><br>Parent Participation*	culture and space-dependent nursing support, and controlled or empowering breastfeeding experiences
Jannes et al.<br>2020<br>[31]<br><br>Germany	Cross sectional<br><br>63%<br><br>Hierarchical mixed-effects logistic models adjusted for confounders	66 NICUs<br><br>Parents and their VLBW infants<br><br>Infants (n=923)<br>Parents (n=1277)	Existing FCC practices were evaluated for the following variables: recreation room, rooming in, unrestricted visiting hours for parents, parental classes, connection to parent associations, and standards on developmentally supportive care<br><br>Respect and Dignity*<br><br>Information Sharing*<br><br>Parent Participation*	Quantitative Results<br><br>Parents<br>Two out of these six variables were significant<br>predictors of parent satisfaction:<br><br>Unrestricted visiting hours<br><br>Standardized procedures for developmentally supportive care.
Khanjari et al.<br>2022<br>[34]	Quasi-experimental comparing pre- and post-	NICUs in two hospitals<br><br>Parents (n=52 pairs of mothers/fathers)<br><br>Infants (n=52)	FCC education sessions 60 minutes x 3 through face-to-face conversation and the use of videos and slides and Simulations. Education about premature infant bathing,	Quasi-Experimental Results<br>Differences from pre- to post:<br><br>Parents<br>QOL

Author<br>Year<br><br>Country<br><br>[Reference]	Study Design<br><br>Quality Appraisal	Setting<br><br>Sample<br><br>Family Involvement	Intervention Description<br><br>FCC Principles<br>(Primary Emphasis*)	RCT or Quasi-Experimental Results[1, 2]<br><br>Quantitative Descriptive Results<br><br>Qualitative Results
 Iran	No comparison group in this study but compared mothers' scores to fathers' scores<br><br>82%<br><br>Independent t-test and paired t-test<br><br>Comparison of characteristics between groups not reported		baby hugging, breastfeeding, nutrition, post-vaccination care, diaper change, umbilical cord care, jaundice and colic care, sleep, and safety.<br><br>Respect and Dignity<br><br>Information Sharing*<br><br>Parent Participation	Fathers' mean total QOL and 4 of 5 of its components increased by 11-17%<br><br>Mothers' mean total QOL and all 5 of its components increased by 12-22%<br><br>Mothers had a larger mean improvement in QOL than fathers<br><br>Worsened<br>QOL<br>Fathers' physical component decreased by 3%
Kidszun et al. 2020<br>[32]<br><br>Germany	Cross sectional<br><br>Descriptive only<br><br>63%	NICU<br><br>Infants<br>(n=67)	Existing FCC in NICU evaluated for VRTI outcomes. FCC included communication, involvement in care, Kangaroo Care, peer-to-peer support, and parent education. No rooming in, but parents had a lounge and separate family home for overnight stays. The	Descriptive Results<br><br>In total, 75 symptomatic screenings and 272 weekly screenings were recorded<br><br>Infants who developed VRTI <3%<br><br>No new VRTI were diagnosed during asymptomatic screenings

Author<br>Year<br><br>Country<br><br>[Reference]	Study Design<br><br>Quality Appraisal	Setting<br><br>Sample<br><br>Family Involvement	Intervention Description<br><br>FCC Principles<br>(Primary Emphasis*)	RCT or Quasi-Experimental Results[1, 2]<br><br>Quantitative Descriptive Results<br><br>Qualitative Results
			NICU was open 24/7 to families and close friends, two at a time. Siblings of all ages welcomed with a health check. Stressed hand hygiene, avoiding visits in cases of respiratory illness, prompt isolation measures for virus-positive cases<br><br>Respect and Dignity<br><br>Information Sharing*<br><br>Parent Participation*	Daily parent visits were seen during 89% of screenings<br>Siblings under 12 years visited in 11% of screenings<br>One or more siblings under age 12 were present in the NICU 40% of the time
Klein et al.<br>2021<br>[35]<br><br>France	Quasi-experimental comparing different groups pre- and post-<br><br>EHR data<br><br>100%	NICU<br><br>EPI < 28 weeks GA<br><br>Infants total (n=228)<br><br>2007–2008 (n = 54) before intervention<br><br>2010–2011 (n = 77) during intervention	NIDCAP, a complex intervention that adjusts to the needs of the infant. Based on neurodevelopment, parent-infant interactions, parent involvement, and breastfeeding promotion<br><br>Respect and Dignity<br><br>Information Sharing	Quasi-Experimental Results<br>Differences between first and third cohorts:<br><br>Infants<br>Number of painful procedures decreased from a mean of 50 to 37<br>Pain evaluations increased from a mean of 3 to 46<br><br>Increased weight gain at discharge by a mean of 359 grams

Author<br>Year<br><br>Country<br><br>[Reference]	Study Design<br><br>Quality Appraisal	Setting<br><br>Sample<br><br>Family Involvement	Intervention Description<br><br>FCC Principles<br>(Primary Emphasis*)	RCT or Quasi-Experimental Results[1, 2]<br><br>Quantitative Descriptive Results<br><br>Qualitative Results
	ANOVA, linear and logistic regression adjusted for confounders	2013–2014 (n = 97) during intervention	Parent Participation*	Received at least one SSC increased from 25% to 87%<br><br>First SSC was performed on average 4 days earlier and lasted twice as long<br><br>Respiratory: duration of mechanical ventilation decreased, magnitude not reported<br><br>Parents<br>Presence increased from a mean of 26 to 40 hours<br><br>No differences<br>Mortality and morbidity, LOS, duration of oxygen supplementation, use of postnatal steroids, ROP, IVH, PVLM, late onset neonatal infection, or duration of parenteral nutrition
Lægtskov et al.<br>2023<br>[48]	Qualitative descriptive	Level 2 NICU<br><br>Fathers of preterm infants<br><br>Fathers total (n=10)	Knowledge sharing groups aimed to develop the family's knowledge, skills, and function through a partnership of HCP and the families. Fathers' groups were 90	Qualitative Results<br><br>The overall theme was "Emotional support, encouragement, and an enhanced capacity to deal with the situation and with life in the NICU"

Author<br>Year<br><br>Country<br><br>[Reference]	Study Design<br><br>Quality Appraisal	Setting<br><br>Sample<br><br>Family Involvement	Intervention Description<br><br>FCC Principles<br>(Primary Emphasis*)	RCT or Quasi-Experimental Results[1, 2]<br><br>Quantitative Descriptive Results<br><br>Qualitative Results
Denmark	Semi-structured interviews<br><br>100%	Infants total (n=14 including 2 sets of twins)	minutes long and facilitated by a neonatologist who is a father himself Participants attended at least one fathers' group.<br><br>Respect and Dignity<br><br>Information Sharing*<br><br>Parent Participation	Subcategories1) To relate one's story to those of others, 2) Not strangers anymore, 3) Reinforced skills and competencies, 4) A safe space with a focus on fathers in the NICU, and 5) The setting for the fathers' group
Lyngstad et al.<br>2022<br>[36]<br><br>Norway	Quasi-experimental comparing different groups pre- and post-<br><br>QI project<br><br>Descriptive only<br><br>82%	Level 2 NICU with 17-bed, SFR<br><br>Infants total (n=89)<br>Pre- (n=37)<br>Post- (n=52)<br><br>Parents total estimate (n=89; states that at least one parent per infant participated)	FCC cultural change intervention included COMFORTneo pain training for staff, facilitating parent participation and presence for painful procedures, pain management guidelines, ergonomic equipment to facilitate parental involvement<br><br>Respect and Dignity<br><br>Information Sharing<br><br>Parent Participation*	Descriptive Results<br>Differences from pre- to post-:<br><br>Parents<br>Participation, 50% vs 82%<br><br>Participation by procedure<br>Venipuncture, 65% vs 87%<br>Insertion of NG tube, 50% vs 73%<br>Insertion of PVC, 0% vs 70%<br>Airway suction, 51% vs 76%

Author<br>Year<br><br>Country<br><br>[Reference]	Study Design<br><br>Quality Appraisal	Setting<br><br>Sample<br><br>Family Involvement	Intervention Description<br><br>FCC Principles<br>(Primary Emphasis*)	RCT or Quasi-Experimental Results[1, 2]<br><br>Quantitative Descriptive Results<br><br>Qualitative Results
Månsson et al.<br>2019<br>[37]<br><br>Sweden	Quasi-experimental comparing different groups pre- and post-<br><br>91%<br><br>Mann–Whitney *U*-test<br><br>Comparison of characteristics between groups not reported	Level 2 NICU<br><br>Parents of preterm infants <37 weeks GA<br><br>Parents total (n=216)<br>Intervention (n=98, n=60 fathers and n=58 mothers)<br>Control (n=118, n=49 mothers and n=49 fathers)<br><br>Infants total (n=122)<br>Intervention (n=58)<br>Control (n=64)	Neonatal Parent Support Program involved four dialogues and daily information exchange between parents and professionals based on parents' needs. Topics 1) reflecting on preterm delivery, infant needs and appearance, 2) how to interpret and interact with infant, 3) communicating about parent's reaction, relationship and future discharge, and 4) summarizing the experiences of the care period<br><br>Respect and Dignity<br><br>Information Sharing*<br><br>Parent Participation	Quasi-Experimental Results<br>Differences from pre- to post:<br><br>NS<br>Parents<br>PSS:NICU<br>Mothers' mean total score difference compared to control group<br>Fathers' mean total score difference compared to control group
Maria et al.<br>2021<br>[33]<br><br>India	Cross sectional<br><br>Feasibility study	NICU in a tertiary hospital<br><br>Parent/infant dyads total (n=333 dyads)<br>Fathers (n=124)	Comprehensive audio-visual training tool with four sequential modules. Parents learned skills and demonstrated competencies.	Descriptive Results<br><br>Planned parent training sessions held (939 of 1242, 76%)

**Author Year**<br><br>**Country**<br><br>**[Reference]**	**Study Design**<br><br>**Quality Appraisal**	**Setting**<br><br>**Sample**<br><br>**Family Involvement**	**Intervention Description**<br><br>**FCC Principles (Primary Emphasis*)**	**RCT or Quasi-Experimental Results**[1, 2]<br><br>**Quantitative Descriptive Results**<br><br>**Qualitative Results**
	Descriptive only<br><br>63%	Mothers (n=320)	This intervention was a paradigm shift in the NICU from parents as passive observers to active willful participants. Topics 1) handwashing, 2) developmental care, hygiene, positioning, nesting, interacting with the infant; breastfeeding, 3) Kangaroo Mother Care, and 4) preparation for discharge and care at home Mothers also received three meals a day at no cost and financial assistance when needed.<br><br>Respect and Dignity<br><br>Information Sharing<br><br>Parent Participation*	Parents completing FCC training sessions<br>All four training sessions, 50%<br>Sessions 1 and 2, 95%<br>Session 3, 60%<br>Session 4, 75%<br>Differences from first to second month:<br><br>Parents<br>Increased by:<br>Maintaining personal hygiene, 29%<br>Handwashing duration, 17%<br>Cleaning baby properly, 6%<br>Breastfeeding positioning, 2%<br>Milk expression technique, 8%<br><br>Decreased by:<br>Handwashing before entering, 22%<br>Handwashing steps, 8%<br>Positioning of infant, 26%<br><br>No differences<br>Removal of accessories, 0% vs 0%<br>Removal of footwear, 0% vs 0%

Mirlashari et al.<br>2021<br>[38]<br><br>Iran	Quasi-experimental two-group study with a pre- vs post-design<br><br>82%<br><br>Independent *t*-test, paired *t*-test, and repeated measure ANOVA<br><br>Potential confounders were comparable between groups	NICU<br><br>Mothers of infants less than 35 weeks GA<br><br>Mothers total (n=80)<br>Intervention (n=40)<br>Control (n=40)	FCC educational intervention. 60 minutes of group discussion intervention x 4 Topics 1) environment and personnel of the NICU, characteristics of the premature infant, the causes of premature birth and routine care, 2) premature infants' needs, environmental stimuli, positioning, Kangaroo mother care, 3) feeding, milk and preservation of the mother's milk, urination/defecation of the infant and changing the diaper and 4) infant's sleep, common problems of a premature infant, jaundice<br><br>Mothers in the control group received routine care, not described<br><br>Respect and Dignity<br><br>Information Sharing*<br><br>Parent Participation	Quasi-Experimental Results<br>Differences from pre- to post:<br><br>Parents<br>Stress: mean total scores decreased by 36% compared to 8% in the control group<br><br>Problem-focused coping (more desirable): mean scores increased by 5% compared to less than 1% in the control group<br><br>Emotion-focused coping (less desirable) mean scores decreased by 4% compared to 1% in the control group
Neu et al.<br>2020<br>[47]<br><br>US	Qualitative<br><br>83%	2 NICUs<br>One NICU in a tertiary Children's hospital and the other a university-based birthing hospital	Existing FCC practices were evaluated. Mother's experiences were compared to the experiences of mothers 2	Qualitative results<br>5 themes that emerged were<br><br>1) Visiting: barriers including birth complications, childcare, and finances

Author<br>Year<br><br>Country<br><br>[Reference]	Study Design<br><br>Quality Appraisal	Setting<br><br>Sample<br><br>Family Involvement	Intervention Description<br><br>FCC Principles<br>(Primary Emphasis*)	RCT or Quasi-Experimental Results[1, 2]<br><br>Quantitative Descriptive Results<br><br>Qualitative Results
		Mothers of infants <32 weeks GA<br><br>Mothers total (n=14) purposive sampling until saturation	decades ago as described in a literature review<br><br>NICU #1 The unit consisted of 74 private rooms and 8 twin rooms with a pull-out bed and a recliner in each room. Both parents could stay overnight with their infant. Nine lactation consultants covered outpatient and inpatient settings.<br><br>NICU #2 was divided into 4 pods, each holding 8 to 12 infants. Each bedspace had a private area that was closed with a curtain. There were 6 twin spaces and 2 "rooming-in" rooms where the infant stayed with parents. A lactation support specialist was available.<br><br>Respect and Dignity<br><br>Information Sharing	Mothers valued a comfortable place to sleep. Most had social support but two mothers who didn't had more negative feedback overall. Social support groups at the hospitals had mixed reviews<br><br>2) General caregiving: mothers expressed anxiety, reported high levels of participation in care, knowledge and growing confidence. Relationships with nurses were mostly positive<br><br>3) Holding: began on day of life 3-10 for the group of mothers. Most valued SSC care and began managing their own holding over time<br><br>4) Feeding: Half the mothers gave their infants breastmilk. They reported good lactation support, some challenges with breastfeeding<br><br>5) Maternal ideas for improvement: the NICU environment, more support groups, more check in from staff about mothers' need for resources

**Author**<br>**Year**<br><br>**Country**<br><br>**[Reference]**	**Study Design**<br><br>**Quality Appraisal**	**Setting**<br><br>**Sample**<br><br>**Family Involvement**	**Intervention Description**<br><br>**FCC Principles**<br>**(Primary Emphasis*)**	**RCT or Quasi-Experimental Results[1, 2]**<br><br>**Quantitative Descriptive Results**<br><br>**Qualitative Results**
			Parent Participation*	
Pillai et al.<br>2022<br>[44]<br><br>India	RCT, two-group study<br><br>60%<br><br>2-sample *t*-test and Mann Whitney test<br><br>Results not adjusted for confounders despite differences between groups	NICU of a tertiary hospital<br><br>Families of infants 28-32 weeks GA<br><br>Parents<br>Intervention group (n=73)<br>Control group (n=74)<br><br>Parents total (n=147)	FCC education intervention. Early parent participation program (EPPP) started on the first or second day of life. Five education modules consisting of audio-visual aids, face-to-face sessions, and participation booklet. Families received bedside coaching and supervision in orogastric tube feeding, cup or bottle feeding, nesting, diaper changing, temperature check, giving oral supplements, SSC, daily weight check. Parents tracked their activities in a booklet<br><br>Control group received routine care<br><br>Respect and Dignity<br><br>Information Sharing*<br><br>Parent Participation*	Quasi-Experimental Results<br>Differences between intervention and control:<br><br>Infants<br>Physiologic instability, 47% vs 66%<br>Median number instability events 0 vs 1<br>Feeding intolerance, 18% vs 36%<br><br>Parents<br>Feel confident in performing PP skills 89% vs 73%<br><br>NS<br>Infants<br>Apnea<br>Sepsis workup<br>Breastfeeding rates at discharge<br>Early discharge<br><br>Secondary outcomes<br>Mortality (0 vs 0), number of unstable events, respiratory outcomes, pressor support, full feeds, ROP, PVL, NEC, LOS, Weight, EUGR at 40 weeks

Author<br>Year<br><br>Country<br><br>[Reference]	Study Design<br><br>Quality Appraisal	Setting<br><br>Sample<br><br>Family Involvement	Intervention Description<br><br>FCC Principles<br>(Primary Emphasis*)	RCT or Quasi-Experimental Results[1, 2]<br><br>Quantitative Descriptive Results<br><br>Qualitative Results
Rajabzadeh et al.<br><br>Iran<br><br>2020<br>[39]<br>Stress of mothers and fathers<br><br>2024<br>[40]<br>PTSD of mothers (retrospective analysis)	Quasi-experimental two-group study with a pre- vs. post-design<br><br>2020<br>82%<br>Paired *t*-test and independent *t*-test<br><br>2024<br>73%<br>Paired *t*-test, independent *t*-test and covariance analysis<br><br>Potential confounders were comparable between groups	NICU of a tertiary hospital.<br><br>Parents of infants 30-37 weeks GA<br><br>2020 Sample<br>Infants total (n=80)<br>Parents total (n=160)<br>Mothers, intervention (n=40)<br>Mothers, control (n=40)<br>Fathers, intervention (n=40)<br>Fathers, control (N=40)<br><br>2024<br>Used mothers' data only	FCC educational intervention included five daily, 60-minute sessions including preterm infant care, participation in infant care, facilitating communication and mutual support of couples, and psychological training on the stress associated with having a preterm infant.<br><br>Respect and Dignity<br><br>Information Sharing*<br><br>Parent Participation	2020 Quasi-Experimental Results<br>Differences from pre- to post-:<br><br>Parents<br>PSS:NICU<br>Mothers' mean total scores decreased by 42% compared to 13% in the control group<br>Fathers' mean total scores decreased by 31% compared to 10% in the control group<br><br>2024 Quasi-Experimental Results<br>Improved mean scores from pre- to post:<br><br>Parents<br>PTSD<br>Mothers' mean scores decreased by 20% compared to 12% in the control group

Author<br>Year<br><br>Country<br><br>[Reference]	Study Design<br><br>Quality Appraisal	Setting<br><br>Sample<br><br>Family Involvement	Intervention Description<br><br>FCC Principles<br>(Primary Emphasis*)	RCT or Quasi-Experimental Results[1, 2]<br><br>Quantitative Descriptive Results<br><br>Qualitative Results
	for both 2020 and 2024			
Rosenthal et al.<br>2021<br>[45]<br><br>US	Pilot RCT with feasibility and exploratory outcomes<br><br>60%<br><br>2:1 randomization<br><br>Chi-square test, *t*-test, and nonparametric equality-of-medians test<br><br>Potential confounders were comparable between groups	NICU<br><br>Infants less than 365 days old<br><br>Mother-infant dyads total (n=110)<br>Intervention (n=74)<br>Control (n=36)	Virtual FCR via Zoom was available for parents to attend Monday-Friday. Parents could participate in virtual FCR as much or as little as they chose. They additionally had the option to participate in FCR in person or to not participate in FCR<br><br>Control group had routine care, which included access to FCR in person or the option not to attend (no virtual option)<br><br>Respect and Dignity<br><br>Information Sharing*<br><br>Parent Participation	RCT Results<br>Differences between intervention and control groups<br><br>Infants<br>Exclusive breastmilk feeding at discharge rate 31% compared to 11% in the control group with a difference of 20.0 CI (3.1–37.0)<br><br>LOS<br>Median LOS 12 vs 20 days with a difference of 8 days Effect size C statistic = 0.62 (0.50–0.75)<br><br>Parents<br>Composite caregiver experience<br>Mean scores were 65% compared to 22% in the control group<br><br>Participation<br>Parents in intervention group had 3.4 times the attendance at FCR vs control group CI (2.7-4.2)

Author<br>Year<br><br>Country<br><br>[Reference]	Study Design<br><br>Quality Appraisal	Setting<br><br>Sample<br><br>Family Involvement	Intervention Description<br><br>FCC Principles<br>(Primary Emphasis*)	RCT or Quasi-Experimental Results[1, 2]<br><br>Quantitative Descriptive Results<br><br>Qualitative Results
				Differences from pre- to post:<br>Stress<br>Mothers' mean total scores decreased by 42% compared to 13% in the control group<br><br>NS<br>Infant<br>Any breastmilk feeding<br>Medical errors<br><br>Descriptive Results<br><br>Three of the five feasibility objectives were met (technical issues, time burden and data collection) and two were not (recruitment and intervention uptake)
Saldanha & Gretta Tauro<br>2023<br>[41]<br><br>India	Quasi-experimental comparing different groups pre- and post-<br><br>Pilot study	NICUs in 2 private hospitals<br><br>Mothers of preterm infants 30–34 weeks GA<br><br>Mother-infant dyads total (n=60)<br>Intervention (n=30)<br>Control (n=30)	Neurodevelopmental, mother-centered care with one-on-one comprehensive teaching about communication and safety, feeding, skin care, prevention of infection, positioning and Kangaroo Mother Care	Quasi-experimental Results<br>Differences from pre- to post:<br><br>Infants<br>PIBBS feeding scores<br>Higher mean increases in scores compared to the control group, magnitude not reported<br><br>Parents

Author<br>Year<br><br>Country<br><br>[Reference]	Study Design<br><br>Quality Appraisal	Setting<br><br>Sample<br><br>Family Involvement	Intervention Description<br><br>FCC Principles<br>(Primary Emphasis*)	RCT or Quasi-Experimental Results[1, 2]<br><br>Quantitative Descriptive Results<br><br>Qualitative Results
	Sequential enrollment, first 30 mothers were the control group<br><br>Observations on days 1, 7, and 14<br><br>73%<br><br>ANOVA, Wilcoxon signed rank test, Pearsons's correlation and MANOVA<br><br>Results not adjusted for confounders despite differences between groups		Control group received regular care and the same timing of observations<br><br>Respect and Dignity<br><br>Information Sharing*<br><br>Parent Participation	Higher mean increases than the control group, magnitude not reported, for all competencies:<br>Communication and safety<br>Feeding<br>Positioning and Kangaroo Care<br>Prevention of infection<br>Skin care<br><br>NS<br>Infants<br>Weight<br>Behavior scores
Sivanandan et al.	Quasi-experimental	Level 2 NICU	The key interventions were 1) adoption of a unit FCC	Quasi-experimental Results

Author<br>Year<br><br>Country<br><br>[Reference]	Study Design<br><br>Quality Appraisal	Setting<br><br>Sample<br><br>Family Involvement	Intervention Description<br><br>FCC Principles<br>(Primary Emphasis*)	RCT or Quasi-Experimental Results[1, 2]<br><br>Quantitative Descriptive Results<br><br>Qualitative Results
2021<br>[42]<br><br>India	comparing different groups at baseline, during and after intervention<br><br>QI project<br><br>64%<br><br>Descriptive and statistical process control	in a tertiary care teaching center.<br><br>Mother-infant dyads total (n= 505)<br><br>Baseline group (n=98)<br>Intervention group (n=258)<br>Postintervention group (n= 149)	protocol with expanded visitation hours, 2) parental education through audio-visual aids, and 3) capacity building through training and peer support. Hands-on training was provided to mothers tailored to their learning capacity and neonatal needs Included handwashing, caregiving activities (diaper-change, orogastric tube feeding and spoon feeding)<br><br>Respect and Dignity<br><br>Information Sharing*<br><br>Parent Participation*	Differences from baseline to intervention to postintervention<br>Statistical process control P chart<br><br>Parents<br>Participation by mothers increased 32% vs 44% vs 66%<br><br>Mothers' participation in Kangaroo Care, baby care and diaper change, orogastric or spoon feeding, magnitude not reported<br><br>No difference<br>Incidence of sepsis
Zhang<br>2022<br>[43]<br><br>China	Quasi-experimental two-group study<br><br>Study design included previous NICU	NICU<br><br>Infants in a palliative care pathway and their parents<br><br>Parent-infant dyads total (n=45)<br>Intervention (n=20)	Parents were allocated into a family supportive EOLC intervention group or a standard EOLC group based on their wishes<br><br>FCC included separated single-bedded EOLC room,	Quasi-experimental Results<br><br>Parents<br>Depression<br>Mothers' mean total scores were 2% lower than control group on EPDS

Author<br>Year<br><br>Country<br><br>[Reference]	Study Design<br><br>Quality Appraisal	Setting<br><br>Sample<br><br>Family Involvement	Intervention Description<br><br>FCC Principles<br>(Primary Emphasis*)	RCT or Quasi-Experimental Results[1, 2]<br><br>Quantitative Descriptive Results<br><br>Qualitative Results
	parent representatives<br><br>91%<br><br>Student *t*-test and Chi-square test<br><br>Results not adjusted for confounders despite differences between groups	Control (n=25)	parents participated in basic care including physical contact with their infant and creating commemorative items. A psychologist supported the parents daily to listen to the concerns of parents and to provide emotional support.<br><br>Respect and Dignity<br><br>Information Sharing<br><br>Parent Participation*<br><br>Collaboration in Development	Fathers’ mean total scores were 3% lower on the HAM-D than the control group<br><br>All parents had 2% higher satisfaction scores on medical treatment, medical staff's negotiation attitude, hospital settings and social service than control group

Table notes
CI, confidence interval; EPDS, Edinburgh postnatal depression scale; EHR, electronic health record; EPI, extremely preterm infant < 28 weeks GA; EUGR, extrauterine growth restriction; FCC, family-centered care; FCR, family-centered rounds; GA, gestational age; HAM-D, Hamilton depression rating scale; HCP, healthcare provider; IVH, intraventricular hemorrhage; LOS, length of stay; NEC, necrotizing enterocolitis; NICU, neonatal intensive care unit; NIDCAP, neonatal individualized developmental care and assessment program; PVL(M), periventricular leukomalacia; RCT, randomized controlled trial; ROP, retinopathy of prematurity; QOL, quality of life; SFR, single family room; SSC, skin-to-skin care; US, United States; VLBW, very low birthweight; VRTI, viral respiratory tract infection
[11]For clarity, we rounded most data to the nearest % or whole number
[2] All are significant (p<.05 or less) unless otherwise noted as descriptive findings; NS=non-significant
*Primary principle of the FCC intervention

Table S4 Modified MMAT Quality Assessment

A. Design: RCT

First Author	2.1. Randomization appropriately performed	2.2. Groups were comparable at baseline	2.3. Complete outcome data	2.4. Outcome assessors were blinded to intervention	2.5. Participants adhered to intervention	Total/ total possible	%
Pillai [44]	1	0	1	0	1	3/5	60%
Rosenthal [45]	1	1	1	0	0	3/5	60%

B. Design: Quasi-experimental

First Author	3.1.a Clearly reports inclusion and exclusion criteria	3.1.b States why some chose not to partici-pate	3.1.c Attempts to represent the target population	3.2.a Measure-ment variables are clearly defined	3.2.b Variables are accurately measured	3.2.c Measure-ments are justified and appro-priate	3.2.d Uses Validated and reliability tested measures	3.3 Outcome data are at least 80% complete	3.4.a Collected data on confoun-ders	3.4.b Analyzed data on confound-ers	3.5 Interven-tion was adminis-tered as intended	Total/ total pos-sible	%
Khanjari [34]	1	1	0	1	1	1	1	1	1	0	1	9/11	82%
Klein [35]	1	1	1	1	1	1	1	1	1	1	1	11/11	100%
Lyngstad [36]	1	1	0	1	1	1	1	1	1	0	1	9/11	82%
Månsson [37]	1	1	1	1	1	1	1	1	1	0	1	10/11	91%
Mirlashari[38]	1	1	0	1	1	1	1	0	1	1	1	9/11	82%
Rajabzedah[39] 2020	1	0	0	1	1	1	1	1	1	1	1	9/11	82%
Rajabzedha[40] 2024	1	0	0	1	1	1	1	1	1	1	0	8/11	73%
Saldanha and Gretta Tauro [41]	1	0	0	1	1	1	1	1	1	0	1	8/11	73%
Sivanandan[42]	1	0	0	1	1	1	1	1	0	0	1	7/11	64%
Zhang [43]	1	1	1	1	1	1	1	1	1	0	1	10/11	91%

C. Design: Quantitative Descriptive

Author	4.1.a Relevant sample source	4.1.b Clear justification of sampling framework	4.1.c Adequate sampling procedure	4.2.a Clearly reports inclusion and exclusion criteria	4.2.b States why some chose not to participate	4.2.c Attempts to achieve a represent-ative sample	4.3.a Variables clearly defined	4.3.b Variables accurately measured	4.3.c Measures justified and appropriate	4.3.d Measures validated and reliability tested
Antinora[30]	1	0	1	0	0	0	1	1	1	0
Dallas [49]	1	0	1	0	0	0	1	1	1	1
Jannes [31]	1	1	1	1	0	0	1	1	1	1
Kidszun [32]	1	0	1	1	1	0	1	1	1	1
Maria [33]	1	0	1	1	1	1	1	1	1	0

Author *continued*	4.3.e Question-naires pre-tested prior to data collection	4.4.a Has a low non-response rate	4.4.b Reasons given for non-response	4.4.c Statistical compen-sation for non-response	4.5.a Clearly stated statistical analysis	4.5.b Justified statistical analysis for research question			Total/ Total possible	%
Antinora[30]	0	0	0	0	1	0			6/16	38%
Dallas [49]	0	0	0	0	1	1			8/16	50%
Jannes [31]	0	0	0	0	1	1			10/16	63%
Kidszun [32]	0	1	0	0	1	0			10/16	63%
Maria [33]	0	1	0	0	1	0			10/16	63%

D. Design: Qualitative

First Author	1.1 Appropriate qualitative approach for the research question	1.2.a Adequate method of data collection	1.2.b Adequate form of data and justification for any changes	1.3 Findings adequately derived from data	1.4 Interpretation of results substantiated by data	1.5 Data sources, collection, analysis, and interpretation are linked	Total/ total possible	%
Dallas [49]	1	1	1	1	1	1	6/6	100%
Holdren [46]	0	0	1	1	1	1	4/6	67%
Lægtskov [48]	1	1	1	1	1	1	6/6	100%
Neu [47]	1	1	1	1	1	0	5/6	83%

E. Design: Mixed Methods

First Author	5.1 Adequate rationale for mixed methods	5.2.a Integrated for a complete picture such as joint display	5.2.b Stated when integration occurred	5.3 Adequately interpreted Meta-inference	5.4 Divergences addressed if needed	5.5 Components have strong qual and quant methods	Total/ total possible	%
Dallas [49]	1	0	1	0	1	0	3/6	50%
